# Cross Talk among Transporters of the Phosphoenolpyruvate-Dependent Phosphotransferase System in Bacillus subtilis

**DOI:** 10.1128/JB.00213-18

**Published:** 2018-09-10

**Authors:** Kambiz Morabbi Heravi, Josef Altenbuchner

**Affiliations:** a Institut für Industrielle Genetik, Universität Stuttgart, Stuttgart, Germany; Rutgers University-Robert Wood Johnson Medical School

**Keywords:** PTS, carbohydrate uptake, phosphotransfer, enzyme II, permease

## Abstract

The phosphoenolpyruvate-dependent phosphotransferase system (PTS) is the main carbohydrate uptake system in Bacillus subtilis. A typical PTS consists of two general proteins, enzyme I (EI) and a histidine-containing protein (HPr), as well as a specific carbohydrate transporter (or enzyme II [EII]), all of which transfer the phosphoryl group from phosphoenolpyruvate to the transported carbohydrate. The specific PTS transporters are formed by multidomain proteins or single-domain subunits. These domains are domain C (EIIC), the transmembrane channel for the carbohydrate transport; domain B (EIIB), the membrane-bound domain responsible for phosphorylation of the carbohydrate; and domain A (EIIA), the mediator between HPr(H15∼P) and EIIB. There are 16 PTS transporters in B. subtilis, 6 of which, i.e., NagP, MalP, MurP, TreP, SacP, and SacX, contain no EIIA domain. Deletion of the single-EIIA-containing transporters showed that there is cross talk between the noncognate EIIA and EIIB domains in PTS. By deletion of all EIIA-containing proteins, strain KM455 (ΔEIIA) was constructed, and the EIIA-containing proteins were individually introduced into the strain. In this way, the PTS transporters of the glucose family, namely, PtsG, GamP, and PtsA (also known as YpqE), enabled growth with maltose, *N*-acetylglucosamine, sucrose, or trehalose as the sole carbon source. Construction of TkmA-EIIA fusion proteins confirmed the probable interaction between the EIIAs of the glucose family of PTS transporters and the EIIA-deficient PTS transporters. Likewise, we have shown that SacX is mainly phosphorylated by PtsA and GamP. PtsG and GmuA were also able to phosphorylate SacX, albeit less well than GamP and PtsA.

**IMPORTANCE** The phosphoenolpyruvate-dependent phosphotransferase system (PTS) not only is a carbohydrate uptake system in B. subtilis but also plays an important role in sensing the nutrient fluctuation in the medium. This sensing system enables the cells to respond to these fluctuations properly. The PTS transporters have a pivotal role in this sensing system since they are carbohydrate specific. In this study, we tried to understand the interactions among these transporters which revealed the cross talk among PTSs. Three PTS proteins, namely, PtsG (the specific transporter of glucose), GamP (the specific transporter of glucosamine), and PtsA (a cytoplasmic single-domain EIIA protein) were shown to play the major role in the interaction among the PTSs.

## Abstract (precis)

The phosphoenolpyruvate-dependent phosphotransferase system (PTS) not only is a carbohydrate uptake system in B. subtilis but also plays an important role in sensing the nutrient fluctuation in the medium. This sensing system enables the cells to respond to these fluctuations properly. The PTS transporters have a pivotal role in this sensing system since they are carbohydrate specific. In this study, we tried to understand the interactions among these transporters which revealed the cross talk among PTSs. Three PTS proteins, namely, PtsG (the specific transporter of glucose), GamP (the specific transporter of glucosamine), and PtsA (a cytoplasmic single-domain EIIA protein) were shown to play the major role in the interaction among the PTSs.

## INTRODUCTION

Carbohydrates are mainly taken up and phosphorylated by transporters (enzyme II [EII]) of the phosphoenolpyruvate (PEP)-dependent phosphotransferase system (PTS) in Bacillus subtilis (1). The PTS transporters are formed by domains or subunits with different functions. The EIIC domain (together with EIID in the levan PTS) forms a transmembrane channel. The EIIB domain phosphorylates the incoming carbohydrate. The EIIA domain carries the phosphoryl group from the histidine-containing protein HPr, a general PTS protein, to EIIB (for reviews, see references 2 and 3). The PTS transporters of B. subtilis are classified into 5 different families, i.e., the glucose, β-glucoside, lactose, mannose, and fructose/mannitol transporter families, based on the phylogeny of the EIIC domain (Table 1). Each family has a different domain structure and arrangement. For instance, the domain arrangement in the glucose family is EIICBA, whereas it is EIIBCA in the β-glucoside family. Notably, among 16 PTS transport systems, 6 transporters, i.e., MalP, MurP, NagP, SacP, SacX, and TreP, contain no EIIA domain (shown by bold letters in Table 1) (4). The operons encoding the PTS transporters are shown in Fig. S1 in the supplemental material.

**TABLE 1:** The encoding genes of the PTS transporters in B. subtilis^*a*^

Gene	Domain structure	Substrate
Glucose family		
*gamP*	EIICBA	GlcN
***nagP***	**EIICB**	**GlcNAc**
***malP***	**EIICB**	**Maltose**
*ptsG*	EIICBA	Glucose
*ypqE*	EIIA	Unknown
*yyzE*	EIIA	Unknown
β-Glucoside family		
***murP***	**EIIBC**	**MurNAc (?)**
***treP***	**EIIBC**	**Trehalose**
***sacP***	**EIIBC**	**Sucrose**
***sacX***	**EIIBC**	**Sucrose**
*vbglP*	EIIBCA	β-glucosides
Lactose family		
*ywbA*	EIIC	Unknown
*licA*	EIIA	Lichenan
*licB*	EIIB	Lichenan
*licC*	EIIC	Lichenan
*gmuB*	EIIB	Mannan
*gmuA*	EIIA	Mannan
*gmuC*	EIIC	Mannan
Mannose family		
*levD*	EIIA	Fructose
*levE*	EIIB	Fructose
*levF*	EIIC	Fructose
*levG*	EIID	Fructose
Fructose/mannitol family		
*mtlA*	EIICB	Mannitol
*mtlF*	EIIA	Mannitol
*manP*	EIIBCA	Mannose
*fruA*	EIIABC	Fructose

a Boldface data indicate the EIIA-deficient PTS transporters in B. subtilis.

MalP is the specific transporter of maltose whose encoding gene is in the *malARP* operon. Maltose is taken up by malP and converted to maltose 6-phosphate, which is then converted to glucose and glucose 6-phosphate by 6-phospho-α-glucosidase (MalA). The *malARP* operon is regulated by MalR (also known as GlvR), which is a transcriptional activator that is thought to interact with maltose 6-phosphate as its effector. Moreover, there is a maltodextrin utilization system in B. subtilis encoded by an ATP-binding cassette (ABC) transport system which is encoded by the *mdxRDEFG-yvdJ-malKL-pgcM* operon. This system mainly takes up maltopentaose and maltohexaose oligosaccharides (5, 6). MurP is the specific transporter of *N*-acetylmuramic acid (MurNAc), which is encoded within the *murQRP-amiE-nagZ-ybbC* operon (7). The *murQRP-amiE-nagZ-ybbC* operon plays an important role in peptidoglycan recycling. So far, most studies on this operon have concerned the catabolic enzymes, whereas the nature of its regulation by MurR remains unknown (8). NagP transports *N*-acetylglucosamine (GlcNAc) and phosphorylates it to produce GlcNAc 6-phosphate. The next step is deacetylation of GlcNAc 6-phosphate by NagA to produce glucosamine 6-phosphate. NagB then converts glucosamine 6-phosphate to fructose 6-phosphate by its deaminase activity (for a review, see reference 9). Expression of *nagP*, *nagABR*, and the *mapB-yflH* operon is regulated by NagR, which is a repressor belonging to the GntR family (10). Both glucosamine 6-phosphate and GlcNAc 6-phosphate specifically bind to NagR and produce structural rearrangements (10, 11).

SacP is the sucrose-specific transporter which takes up sucrose and converts it to sucrose 6-phosphate (12). Sucrose 6-phosphate is then hydrolyzed by an endocellular sucrose, 6-phosphate hydrolase SacA, to fructose and glucose 6-phosphate (13, 14). Fructose can be phosphorylated by intracellular fructokinase GmuE or diffuse outside the cell and can later be taken up by LevDEFG or FruA (1, 15). The *sacP* gene is located within *sacPA-ywdA* operon, which is regulated by a PTS regulation domain (PRD)-containing antiterminator, SacT (7, 16, 17). The PRD-containing regulators contain special PRDs whose phosphorylation activates or deactivates these proteins. Usually, phosphorylation of the PRDII domain by HPr(H15∼P) activates these regulators, whereas phosphorylation of the PRDI domain by the cognate-specific transporter deactivates them. Notably, other PRD-containing regulators, such as MtlR, contain domains other than PRDs, called EIIA- and EIIB-like domains. In the latter case, the interaction between the specific transporter and regulator mainly takes place via the EIIA- and EIIB-like domains (for a review, see reference 18). SacX is another PTS transporter that is thought to transport sucrose; however, this protein is mainly a sucrose sensor since the cells lacking *sacX* are able to grow efficiently with sucrose. The *sacX* gene is located in a bicistronic operon consisting of *sacX* and *sacY* (7, 19). Similar to SacT, SacY is also a PRD-containing antiterminator regulating the expression of the *sacXY* operon as well as the *sacB-levB-yveA* operon (7, 17, 20, 21). SacB and LevB are involved in formation and degradation of levan (22, 23). SacT and SacY are activated at low and high concentrations of sucrose, respectively. It was previously shown that SacT is able to regulate the expression of *sacB-levB-yveA* more strongly than SacY (24). In contrast, there are other reports indicating that the expression of the *sacPA* operon is regulated by SacY, whereas SacT has no influence on the *sacB-levB-yveB* operon (25). TreP is the specific transporter of trehalose whose activity results in the uptake and phosphorylation of trehalose (26). TreA, which is a phospho α-(1,1)-glucosidase, then converts trehalose 6-phosphate to glucose and glucose 6-phosphate (27). The released glucose can be further phosphorylated by glucokinase (GlcK) to glucose 6-phosphate (28) or diffused to the medium and then taken up by the glucose uptake systems (29). The *trePAR* operon is regulated by its specific regulator, TreR, which is a repressor belonging to the GntR family of regulators (7, 30, 31). TreR activity is regulated by its effector, trehalose 6-phosphate (30). In addition to the EIIA-deficient PTS transporters, there are two cytoplasmic EIIA-encoding genes found in the genome of B. subtilis, namely, *ypqE* and *yyzE* (4). The functions of these two proteins were unknown prior to this study.

So far, several studies have been carried out to find the possible solution for the problem of phosphorylation of EIICB proteins. However, they led to conflicting observations. For instance, single deletion of *ptsG* increased the generation time of cell growth with trehalose and maltose (4, 32), although another report claimed that either *ptsG* is not required or *ptsG* deletion does not affect the maltose transport (5). Besides, the *gamP* or *ypqE* single mutants grew similarly to the wild-type (wt) strain in the presence of maltose (4). Also, single deletion of *gamP* and *ypqE* was shown to increase the doubling time of the B. subtilis growth with GlcNAc, while *ptsG* had no effect on the growth with GlcNAc (4). Triple deletion of the *ptsG*, *gamP*, and *ypqE* genes was also reported to increase the doubling time seen with GlcNAc (33). Interestingly, PtsG from B. subtilis was reported to be necessary for phosphorylation of sucrose by EIICB^Sac^ *in vitro* (34). The aim of this study was to find the specific EIIA domains (or proteins) phosphorylating the EIIB domains of MalP, NagP, SacP, SacX, or TreP. Here, we showed that there is cross talk among the PTS transporters of B. subtilis where the EIIA domains of the glucose family of PTS transporters play the pivotal role in the phosphorylation of EIIA-deficient PTS transporters.

## RESULTS

### Cross talk between PTS transporters enables B. subtilis to grow with maltose, GlcNAc, sucrose, or trehalose as the sole carbon source.

**FIG 1:**
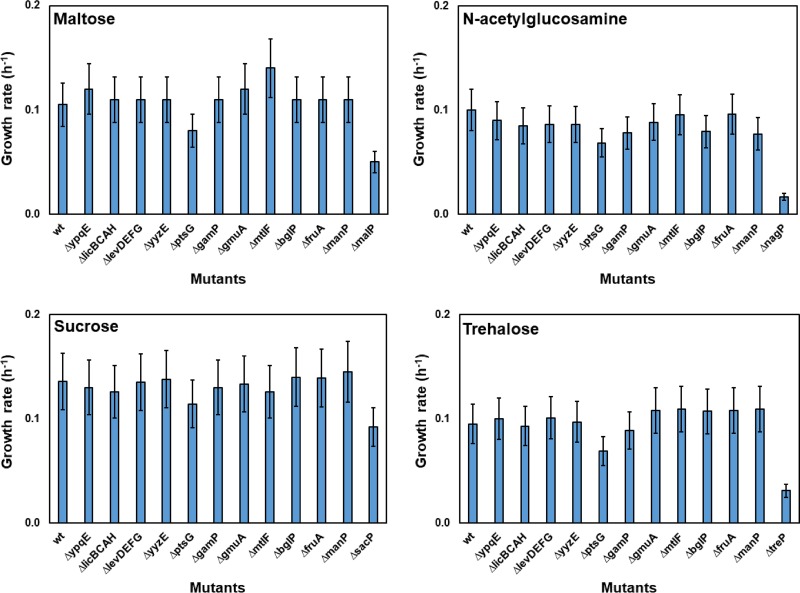
Deletion of the EIIA-encoding genes. The average growth rate of strains KM272 (Δ*ypqE*), KM285 (Δ*licBCAH*), KM287 (Δ*levDEFG*), KM288 (Δ*yyzE*), KM364 (Δ*ptsG*), KM366 (Δ*gamP*), KM373 (Δ*gmuA*), KM358 (Δ*mtlF*), KM423 (Δ*bglP*), KM435 (Δ*fruA*), and KM646 (Δ*manP*) was investigated by the use of Spizizen's minimal medium without citrate containing 0.5% (wt/vol) of maltose, GlcNAc, sucrose, or trehalose as the sole carbon source. Strain KM0 (wt) was used as the positive control, while strains KM402 (Δ*manPA* Δ*mdxRDEFG-yvdJ-malKL-pgcM* Δ*malP*), KM418 (Δ*nagP*), KM422 (Δ*sacP*), and KM338 (Δ*manPA* Δ*treP*) were used as negative-control strains for maltose, GlcNAc, sucrose, and trehalose, respectively. Measurements were carried out at 4-h intervals, and the mean values from three replicates as well as standard deviations (error bars) are demonstrated.

To identify the EIIA domain(s) phosphorylating the EIIA-deficient MalP, NagP, SacP and TreP, the EIIA-encoding genes were individually deleted. The growth of these single-deletion mutants was then measured in minimal media with maltose, GlcNAc, sucrose, or trehalose as the sole carbon source (see Fig. S2 in the supplemental material), and the average growth rate (μ_avg_) of each strain was calculated (Fig. 1). In the minimal medium with maltose, all the single-deletion mutants grew similarly to the wild-type strain (KM0) except the Δ*ptsG* mutant (KM364), which showed a reduced growth rate (Fig. 1; see also Fig. S2). The Δ*malP* mutant (negative control) was unable to grow with maltose after 24 h (Fig. 1; see also Fig. S2). Similar results were obtained when the strains were cultured in the minimal media with GlcNAc or trehalose (Fig. 1). In both of the latter cases, only the Δ*ptsG* mutant grew slower than the wild-type strain, whereas the Δ*nagP* and Δ*treP* mutants (negative controls) were unable to grow with GlcNAc and trehalose, respectively (Fig. 1; see also Fig. S2).

To construct the strain unable to grow with sucrose as the sole carbon source (negative control), *sacP* and *sacX* were deleted alone or together. While deletion of *sacX* had no influence on the growth of the cells with sucrose, deletion of *sacP* (strain KM422) resulted in weaker growth (Fig. S3). Most of the single deletions of the EIIA-encoding genes had no influence on the growth of the strains with sucrose (Fig. 1; see also Fig. S2). Only the Δ*ptsG* mutant grew slower than the wild-type strain (Fig. 1; see also Fig. S2). Taken together, these results indicated that although the deletion of *ptsG* negatively influenced the growth of the cells with maltose, GlcNAc, sucrose, and trehalose, the Δ*ptsG* cells were still able to grow better than the Δ*malP*, Δ*nagP*, Δ*sacP*, and Δ*treP* cells. Therefore, we assumed that EIIA domains other than EIIA^Glc^ could also phosphorylate the EIIA-deficient PTS transporters.

### Introduction of the glucose family of PTS transporters enables the ΔEIIA strain to grow with maltose, GlcNAc, sucrose, and trehalose.

**FIG 2:**
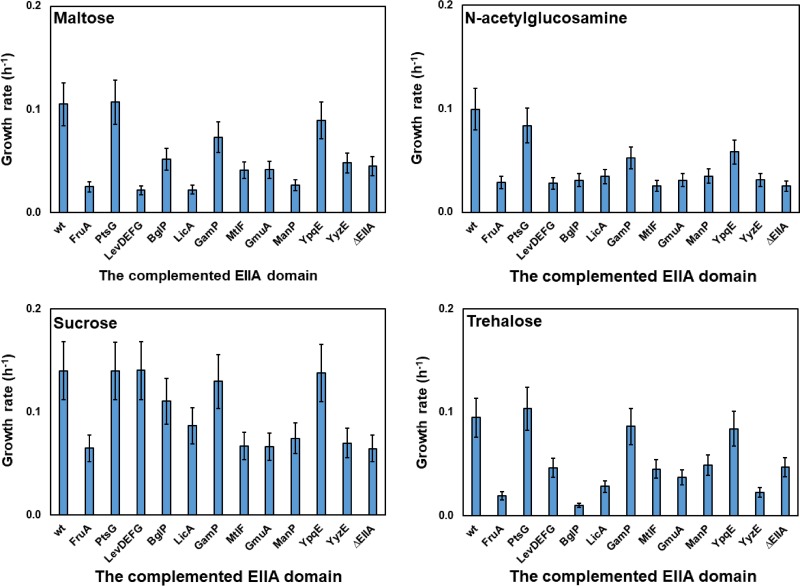
Complementation of the EIIA-deficient PTS transporters with a single EIIA domain in ΔEIIA deletion strain KM455. The average growth rate of strains KM453 (KM455 *fruA*^+^), KM790 (KM455 *ptsG*^+^), KM791 (KM455 *levDEFG*^+^), KM792 (KM455 *bglP*^+^), KM793 (KM455 *licA*^+^), KM794 (KM455 *gamP*^+^), KM795 (KM455 *mtlF*^+^), KM796 (KM455 *gmuA*^+^), KM797 (KM455 *manP*^+^), KM801 (KM455 *ypqE*^+^), and KM802 (KM455 *yyzE*^+^) was investigated. Strain KM0 (wt) was used as the positive control, while strain KM455 (ΔEIIA) was used as the negative control. The experiment was carried out as explained in the legend to Fig. 1.

To study the effect of each EIIA domain on the growth of B. subtilis, all the EIIA-encoding genes were deleted to construct strain KM455 (or ΔEIIA). The ΔEIIA strain was unable to grow with maltose, GlcNAc, sucrose, or trehalose as the sole carbon source (Fig. 2; see also Fig. S4). Different EIIA-encoding genes were then introduced into the ΔEIIA strain, and the growth of each strain containing a single EIIA domain with maltose, GlcNAc, sucrose, and trehalose was studied. Only the presence of PtsG, YpqE, or GamP in the ΔEIIA strain supported the growth of the cells with maltose (Fig. 2; see also Fig. S4). Introduction of other EIIA-containing proteins resulted in a growth rate similar to that of the ΔEIIA strain (Fig. 2; see also Fig. S4). PtsG, YpqE, and GamP also supported the growth of the ΔEIIA strain with GlcNAc and trehalose (Fig. 2; see also Fig. S4). These results, taken together, indicate that PtsG, YpqE, and GamP, all of which belong to the glucose family of PTS transporters in B. subtilis, significantly supported the growth of the ΔEIIA strain with maltose, GlcNAc, and trehalose. In the presence of sucrose, the presence of not only PtsG, YpqE, and GamP but also BglP, LicBCA, or LevDEFG in the ΔEIIA mutational background restored the growth of the cells (Fig. 2; see also Fig. S4).

**FIG 3:**
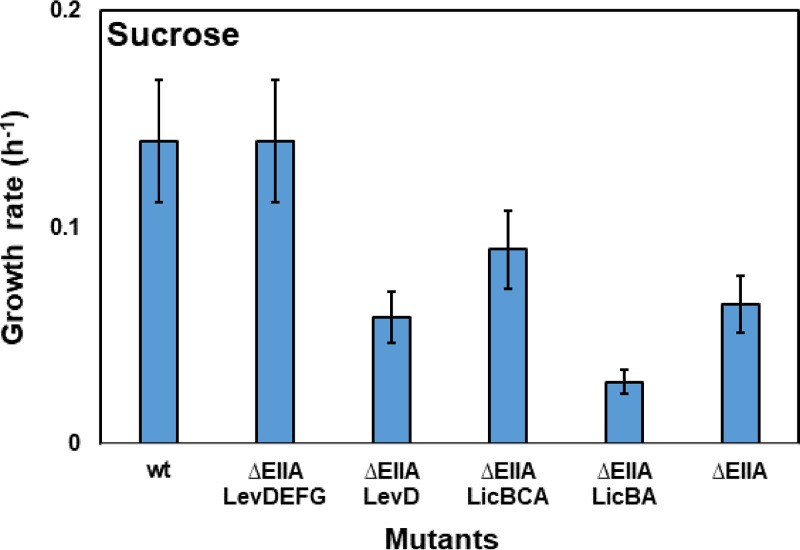
Disabling sucrose transport via LevDEFG and LicBCA complexes. The average growth rate of strains KM791 (KM455 *levDEFG*^+^), KM815 (KM455 *levD*^+^), KM793 (KM455 *licBCA*^+^), and KM820 (KM793 Δ*licC*) was investigated. Strains KM0 (wt) and KM455 (ΔEIIA) were used as positive and negative controls, respectively. The experiment was carried out as explained in the legend to Fig. 1.

On the one hand, all the restored growth could have been due to the phosphoryl transfer from the introduced EIIA domain to the EIIA-deficient PTS transporter. On the other hand, with the exception of YpqE, which is a single-EIIA protein, all of the proteins are multidomain transporters that might be able to transport the carbohydrates nonspecifically. To rule out the latter possibility, the transmembrane domains (EIIC) of LevDEFG and LicBCA were deleted to prevent the sucrose transport. The ΔEIIA strain with only LevD or LicBA was unable to grow in sucrose minimal medium, and the growth rates of the strains were reduced (Fig. 3; see also Fig. S5). This confirmed the possible nonspecific transport of sucrose via the LevDEFG and LicBCA transporters (Fig. 3). In contrast to LevDEFG and LicBCA, deactivation of the carbohydrate transport via PtsG, GamP, and BglP simply by deletion of their transmembrane domain (EIIC) was not possible since all of their PTS domains (EIIC, EIIB, and EIIA) formed a single protein. Therefore, it was necessary to determine whether or not domain separation of the multidomain transporters affects their function. This was investigated using PtsG as a model.

### Separation of the PtsG (EIICBA^Glc^) domains significantly hampered bacterial growth with glucose.

**FIG 4:**
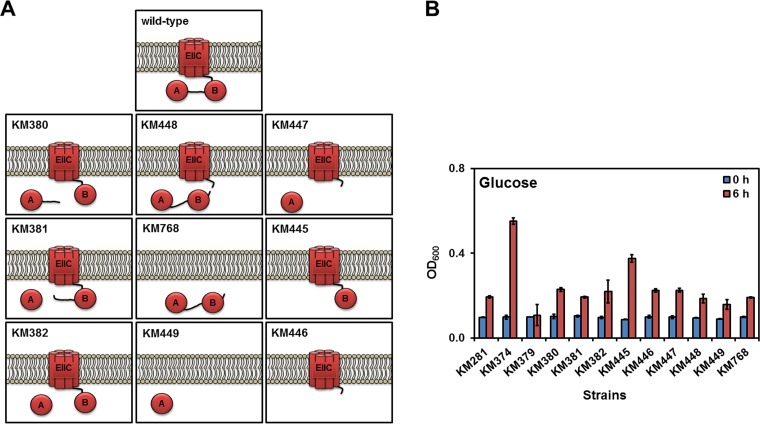
Separation of the PtsG (EIICBA^Glc^) domains. (A) Schematic presentation of the domain structure in the PtsG variants. All *ptsG* variants were expressed under the control of P_*ptsG*_. (B) Growth of the derivatives of strain KM379 (Δ*glcK* Δ[*ptsG*-P_*ptsHI*_-*ptsH*]::*ermC*) carrying a variation of PtsG with truncated or deleted domains was measured in Spizizen's minimal medium without citrate containing 0.5% (wt/vol) of glucose as the sole carbon source. Strains KM281 (Δ*glcK* Δ*ptsG*) and KM379 (Δ*glcK* Δ[*ptsG*-P_*ptsHI*_-*ptsH*]::*ermC*) were used as negative controls, whereas KM374 (Δ*glcK*) was a positive control. The bacterial cultures were started with an OD_600_ of 0.1 in shaking flasks, and growth was measured after 6 h. All experiments were performed as triplicates, and the mean values and standard deviations (error bars) are shown.

In general, glucose is taken up by PTS transporter PtsG (EIICBA^Glc^) or by the non-PTS glucose/mannose:H^+^ symporter (GlcP) during vegetative growth as well as by GlcU during sporulation (35–37). The glucose transported via the non-PTS pathway is phosphorylated inside the cytoplasm by glucose kinase (GlcK) (28). Therefore, to study the transport capability of the PtsG variants (Fig. 4A), it was necessary to construct a strain in which the non-PTS pathway of glucose transport was inhibited. To do so, the *ptsG* alleles were integrated in a Δ*glcK* background (strain KM374) under the control of their wild-type promoter (Fig. S6). In the first step, the *ptsG* gene together with its downstream region containing the promoter of *ptsHI* was replaced with an erythromycin resistance gene in strain KM374 to construct KM379 (Fig. S6). Therefore, KM379 was unable to grow with PTS carbohydrates, including glucose (Fig. 4B). The truncated *ptsG* variants encoding separated or fused EIIC^Glc^, EIIB^Glc^, and EIIA^Glc^ proteins were then integrated with the P_*ptsHI*_-*ptsH* region into KM379 to select the desired strain on minimal plates with PTS carbohydrates (except glucose) and erythromycin sensitivity (Fig. S6). Next, the function of these variants was investigated by the cultivation of strains in minimal media with glucose as the sole carbon source (Fig. 4B). As a negative control, strain KM281 lacking both *ptsG* and *glcK*, albeit with a functional PTS, was used. While strain KM379 did not grow with glucose, strain KM281 showed a doubling after 6 h (Fig. 4B). This indicated that although the glucose uptake systems (PTS and non-PTS) were defective, the cells were still able to take up glucose, likely via the relaxed specificity of other PTS transporters. Therefore, all measurements were carried out after 6 h of cultivation. As a positive control, strain KM374 (Δ*glcK*) harboring the *ptsG* gene was grown until an optical density at 600 nm (OD_600_) of 0.5 was reached under the aforementioned experimental conditions (Fig. 4B).

The EIIA^Glc^ domain was separated from the membrane-anchored EIICB^Glc^ with and without the linker sequence between EIIA^Glc^ and EIICB^Glc^ (strains KM380, KM381, and KM382; Fig. 4A). Strains KM380, KM381, and KM382 grew similarly to strain KM281 (Δ*glcK* Δ*ptsG*). Separation of EIIBA^Glc^ from the EIIC^Glc^ domain in strain KM448 also resulted in weak growth similarly to strain KM281 (Δ*glcK* Δ*ptsG*). Moreover, lack of EIIC^Glc^ (strain KM768), EIIB^Glc^ (strain KM447), EIICB^Glc^ (strain KM449), and EIIBA^Glc^ (strain KM446) hampered the growth with glucose as the sole carbon source (Fig. 4B). Only strain KM445 lacking EIIA^Glc^ could grow with glucose, albeit it did so less well than KM374 (positive control). Taking the data together, this experiment showed that separation of the PtsG domains probably significantly impaired the function of the PtsG domains, especially the phosphoryl transfer. Because these *ptsG* variants were expressed from their natural promoter, any changes in the PtsG activity also affected the *ptsG* expression due to the changes in the phosphorylation status of GlcT, the specific regulator of *ptsG*. Principally, EIIA^Glc^ and EIIB^Glc^ are membrane-bound domains and their expression as cytoplasmic protein could result in their malfunction. To clarify whether the separated EIIBA^Glc^ or EIIA^Glc^ domains remain functional when they are anchored to the cytoplasmic membrane, both variations were expressed under the control of P_*hyperspank*_.

### The membrane-bound EIIA^Glc^ domain remained functional.

**FIG 5:**
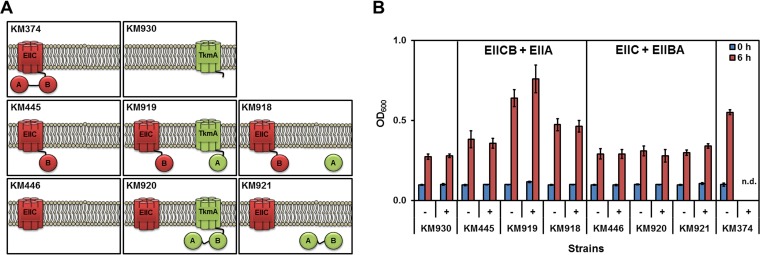
Expression of the EIIBA^Glc^ and EIIA^Glc^ domains as membrane-bound or cytoplasmic proteins. (A) A schematic view of the TkmA-fused proteins and PtsG separated domains is shown. All strains contained the Δ*glcK* mutation. Strain KM374 harbored wild-type *ptsG*, while KM930 lacked *ptsG*. Strains KM918 and KM919 were the derivatives of KM445 producing EIICB^Glc^, whereas KM920 and KM921 were derivatives of KM446 with an EIIC^Glc^ mutational background. The KM919 and KM920 strains expressed TkmA-bound EIIA^Glc^ and EIIBA^Glc^ domains, while KM918 and KM921 expressed cytoplasmic EIIA^Glc^ and EIIBA^Glc^ domains. The proteins expressed under the control of P_*ptsG*_ are shown in red, whereas proteins expressed under the control of IPTG-inducible P_*hyperspank*_ are shown in green. (B) The growth of the strains in minimal medium with glucose as the sole carbon source in shaking flasks was measured after 6 h. After the cells were washed with basal medium, the cells were inoculated with a starting OD_600_ of 0.1. IPTG was added upon inoculation to reach a final concentration of 1 mM. The growth of KM374 in the presence of IPTG was not measured (n.d., no data). All measurements were carried out three times, and the mean values and standard deviations (error bars) are shown.

In a previous study, it was shown that the EIIB^Mtl^ domain of MtlA (mannitol PTS transporter) was not functional when it was overproduced as a cytoplasmic protein. Interestingly, fusion of the EIIB^Mtl^ domain to the transmembrane domains of TkmA, the modulator of tyrosine kinase PtkA (38), restored the activity of EIIB^Mtl^ (39). Here, we used the same strategy with TkmA lacking 34 residues from its C terminus. The EIIBA^Glc^ or EIIA^Glc^ domains were fused to TkmA using His_6_ as a linker (Fig. 5A). The *tkmA-his*_6_-(EIIBA^Glc^/EIIA^Glc^) cassette was expressed under the control of P_*hyperspank*_, and the construct was integrated into the *bglS* locus. The growth of the cells carrying variable constructs were measured in minimal media with and without 1 mM IPTG (isopropyl-β-d-thiogalactopyranoside) as the inducer of P_*hyperspank*_. In order to facilitate the growth of the cells with glucose, two types of overnight cultures (induced and uninduced) were prepared. All strains used in this experiment lacked Δ*glcK* to inhibit the non-PTS glucose uptake system. Strain KM930, the derivative of KM281 (Δ*glcK* Δ*ptsG*) carrying TkmA-His_6_, was used as the negative control, and the results showed a doubling after 6 h of cultivation regardless of the presence or absence of IPTG (Fig. 5B). KM374, the positive-control strain, carrying the wild-type *ptsG* locus was able to grow until an OD_600_ of 0.55 was reached after 6 h of growth (Fig. 5B). In the first step, expression of EIIA^Glc^ was investigated as a membrane-bound protein (KM919) compared with the free cytoplasmic protein (KM918). While strain KM919 grew until an OD_600_ of 0.76 was reached, strain KM918 grew until an OD_600_ of 0.46 was reached (Fig. 5B). Strain KM445, the parental strain of KM918 and KM919, showed growth similar to that seen with the negative control, strain KM930 (OD_600_ of 0.38) (Fig. 5B). This indicated that the membrane-bound EIIA^Glc^ was functional, whereas the cytoplasmic EIIA^Glc^ was almost entirely defective. Next, the EIIBA^Glc^ domains were separated from EIIC domain, and analysis of their expression as a TkmA-fused protein (KM920) or cytoplasmic protein (KM921) was carried out. Strains KM920 and KM921 grew similarly to the parental strain, KM446 (Fig. 5B). Overall, these results indicated that only the membrane-bound EIIA^Glc^ effectively supported the growth with glucose, while its production as a cytoplasmic protein or together with the EIIB^Glc^ domain resulted in defective or weakly functional proteins.

### The EIIA^Glc^, EIIA^Gam^, and EIIA^*ypqE*^ domains restored the growth of the ΔEIIA strain with maltose, GlcNAc, sucrose, and trehalose.

**FIG 6:**
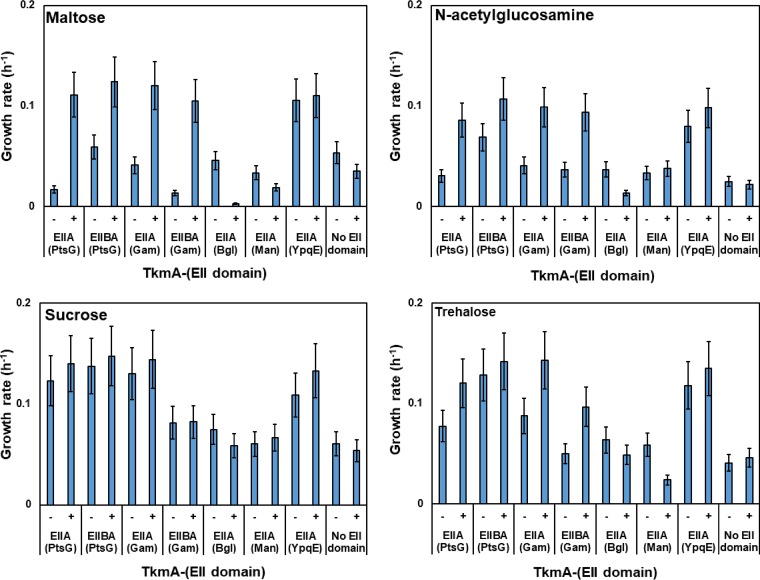
Interaction between the TkmA-fused proteins and the EIIA-deficient PTS transporters. The average growth rate of the ΔEIIA strain containing TkmA-fused proteins in the presence of maltose, GlcNAc, sucrose, and trehalose is shown. Strains KM870 (TkmA-His_6_-EIIBA^Glc^), KM884 (TkmA-His_6_-EIIBA^Gam^), KM885 (TkmA-His_6_-EIIA^Bgl^), KM886 (TkmA-His_6_-EIIA^Man^), KM887 (TkmA-His_6_-EIIA^YpqE^), KM873 (TkmA-His_6_), KM916 (TkmA-His_6_-EIIA^Glc^), and KM917 (TkmA-His_6_-EIIA^Gam^) were cultured in Spizizen's minimal medium without citrate containing 0.5% (wt/vol) of maltose, GlcNAc, sucrose, or trehalose as the sole carbon source. The experiment was carried out as explained in the legend to Fig. 1, and IPTG was added upon inoculation to reach a final concentration of 1 mM.

To prove that the EIIA (or EIIBA) domains of PtsG, GamP, and BglP could phosphorylate the EIIA-deficient PTS transporters, they were fused to TkmA. As a negative control, the EIIA domain of ManP was also fused to TkmA since ManP was unable to restore the growth of the ΔEIIA strain in the minimal media with maltose, GlcNAc, sucrose, or trehalose (Fig. 2; see also Fig. S4). Culturing the ΔEIIA derivatives containing different TkmA-fused proteins indicated that the EIIA domains of PtsG and GamP were able to restore the growth of the strains in minimal media (Fig. 6; see also Fig. S7). In the maltose minimal media, this growth was highly dependent on the induction of P_*hyperspank*_. No growth was observed with all four sugars when *tkmA* (negative control), *tkmA*-′*manP* (EIIA), and *tkmA*-′*bglP* (EIIA) were present in the cells (Fig. 6; see also Fig. S7). This result could have been due either to the lack of phosphoryl transfer from the fusion proteins or to the lack of properly folded proteins. YpqE also strongly supported the growth of the cells with maltose, GlcNAc, sucrose, and trehalose when it was bound to membrane regardless of the presence or absence of IPTG. Altogether, these results clearly pointed out that the EIIA domains of the glucose family of PTS transporters, namely, PtsG, GamP, and YpqE, transfer the phosphoryl group to EIIB^Mal^, EIIB^Nag^, EIIB^Sac^, and EIIB^Tre^.

### EIIB^SacX^ is mainly phosphorylated by EIIA^Gam^ and EIIA^YpqE^.

**FIG 7:**
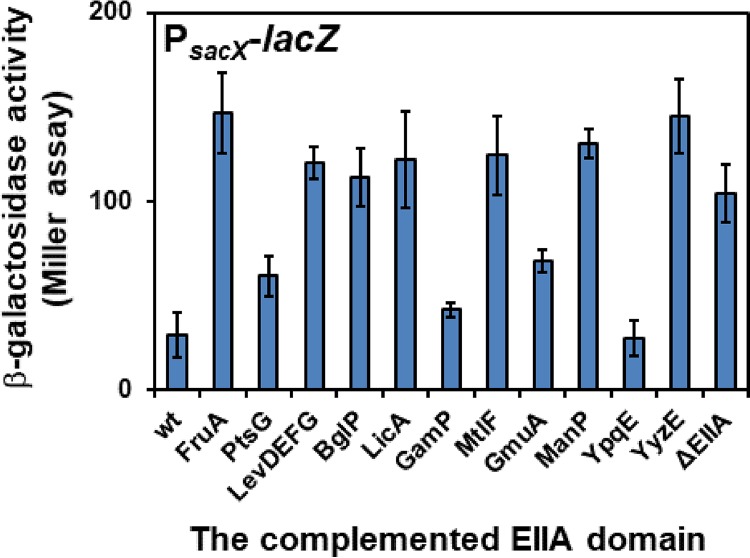
Activity of the *sacX* promoter in the presence of a single-EIIA-containing protein. P_*sacX*_-*lacZ* was integrated into the *amyE* locus of the KM0 wild-type strain and the derivatives of KM455 (ΔEIIA) containing a single-EIIA-containing protein. Strains KM932 (wt), KM933 (FruA), KM934 (PtsG), KM935 (LevDEFG), KM936 (BglP), KM937 (LicA), KM938 (GamP), KM939 (MtlF), KM940 (GmuA), KM941 (ManP), KM942 (YpqE), KM943 (YyzE), and KM944 (ΔEIIA) were used. The β-galactosidase activity of the strains was measured after 3 h of cultivation. Measurements were carried out in triplicate, and the mean values as well as standard deviations (error bars) are shown.

One of the challenges in finding the phosphoryl donor or EIIA domain, which phosphorylates the EIIB domain of SacX, was the inability of SacX to support the growth of B. subtilis with sucrose as the sole carbon source (Fig. S3). Therefore, in order to identify the phosphoryl donor of SacX, another strategy was based on the regulation of *sacX* by its PRD-containing antiterminator, SacY. PRD regulators are regulated by the phosphorylation of their PRD, EIIA-like, and EIIB-like domains by HPr (H15∼P) as well as their cognate transporter. The phosphorylation of PRD-containing regulators by their cognate transporter deactivates them. Therefore, the phosphorylation of SacY by SacX in the absence of sucrose deactivates SacY. In contrast, in the presence of sucrose, SacX dephosphorylates SacY and activates it. Consequently, in the absence of the EIIA domain responsible for phosphorylation of EIIB^SacX^, the SacY antiterminator is active. Using this principle, the promoter region of *sacX* was placed in front of *lacZ* and the activity of P_*sacX*_ was measured in different mutational backgrounds containing a single EIIA domain. In practice, the levels of β-galactosidase activity of the P_*sacX*_-*lacZ* cassette-containing strains were measured after 3 h of inoculation in LB medium without sucrose. As expected, in the wild-type strain, the activity of P_*sacX*_ was one-third of its activity in the ΔEIIA strain (Fig. 7). This shows that SacY is active in the ΔEIIA strain due to the absence of phosphorylation of EIIB^SacX^. In the presence of PtsG, GamP, GmuA, and YpqE, the β-galactosidase activity was similar to that the wild-type strain, probably due to the phosphoryl transfer to the EIIB^SacX^ and deactivation of the SacY as a result.

### PtsA (YpqE) is a PTS EIIA protein whose expression is independent of the PTS carbohydrates.

We have shown that expression of *ypqE* enabled the cells to grow with maltose, GlcNAc, sucrose, and trehalose. Nevertheless, phosphorylation of YpqE by the PTS general proteins, EI and HPr, has not been demonstrated so far. Hence, enzyme I, HPr protein, and YpqE were overexpressed in Escherichia coli strain JW2409-1 lacking its enzyme I (40). After purification of the PTS general proteins and YpqE, an *in vitro* phosphorylation assay was carried out using pyruvate kinase and [γ-^32^P]ATP as the phosphoryl source. The results indicated that YpqE is phosphorylated by HPr(H15∼P) (Fig. S8). Thereafter, *ypqE* was renamed *ptsA*.

**FIG 8:**
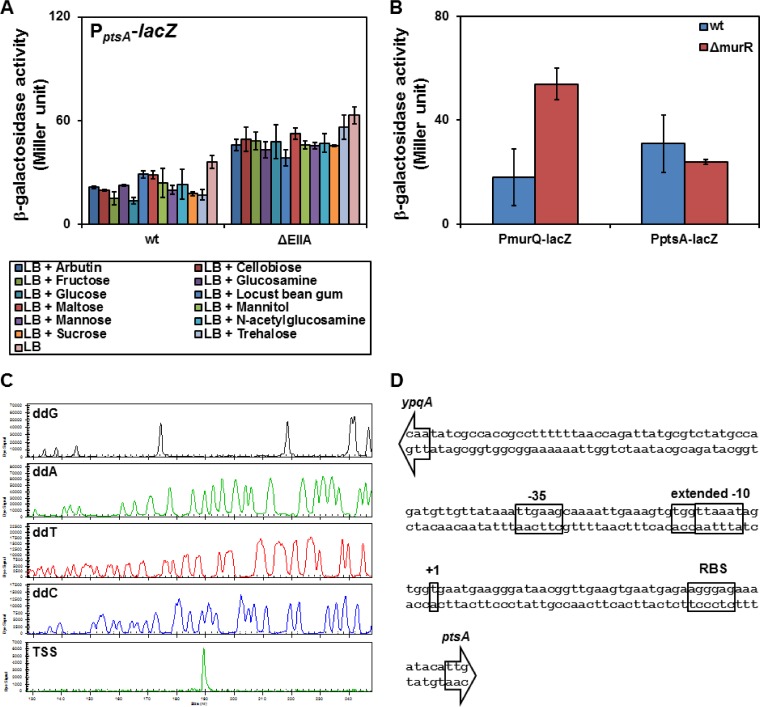
Characterization of the P_*ptsA*_ promoter and its regulation. (A) Activity of the P_*ptsA*_ with different carbohydrates was investigated in the KM679 (wt) and the KM822 (ΔEIIA) strains carrying P_*ptsA*_-*lacZ* integrated in their *amyE* locus. The bacterial culture was inoculated with a starting OD_600_ of 0.05. After 2 h of incubation at 37°C, 0.2% of the desired carbohydrates was added and the β-galactosidase activity in the bacteria was measured after an hour. (B) Studying the activity of MurR, the regulator of the putative MurNAc PTS. The levels of β-galactosidase activity of strains KM679 (wt) and KM849 (Δ*murR*) carrying P_*ptsA*_-*lacZ* (pKAM292) in the *amyE* locus were compared with the levels seen with strains KM877 (wt) and KM878 (Δ*murR*) carrying P_*murQ*_-*lacZ* (pKAM403) in the *amyE* locus. Each strain was inoculated into LB with a starting OD_600_ of 0.05. The β-galactosidase activity was measured after 3 h of incubation. All of the experiments described for panels A and B were carried out in triplicate, and the mean values were used for analysis. The error bars demonstrate the standard deviations. (C) Identification of the transcription start site of *ptsA* by primer extension. The sequencing reaction of the P_*ptsA*_ region on pKAM299 was carried out using 4 separate reaction mixtures, each containing one of the fluorescent-bound dideoxy nucleotides (ddG, ddA, ddT, or ddC), using the s8484 Cy5-labeled oligonucleotide. The primer extension reaction was carried out using the s8484 oligonucleotide and the total RNA isolated from strain KM455 pKAM299. (D) The P_*ptsA*_ sequence containing its core elements (−35 and −10 boxes), the transcription start site (+1), and the ribosomal binding site (RBS). The start codon of *ptsA* is indicated by an arrow.

To better understand the physiological importance of PtsA, regulation of its encoding gene was studied by fusion of the *ptsA* promoter region to *lacZ*. Integration of the P_*ptsA*_-*lacZ* cassette into the chromosome of the KM0 strain (wt) at the *amyE* locus was followed by cultivation of the constructed KM679 strain in LB with all PTS sugars. Measurement of the β-galactosidase level revealed that none of the PTS sugars were able to induce P_*ptsA*_ (Fig. 8A). Next, the P_*ptsA*_-*lacZ* cassette was integrated into the chromosome of KM455 lacking all EIIA domains to construct strain KM822. Due to the absence of EIIA domains in KM822, all the transport systems are dephosphorylated, resulting in activation of PTSs regulated by PRD-containing regulators. Interestingly, the β-galactosidase activity in this strain was doubled compared with the level seen with KM679 in the presence of all PTS sugars (Fig. 8A). None of the global regulators, such as CggR and CcpA, influenced the activity of P_*ptsA*_ (data not shown). The only remaining PTS which was not studied was the putative PTS of MurNAc. So far, the regulation of this system has not been studied. Therefore, the promoter region of *murQ* was placed in front of *lacZ* and the production of the β-galactosidase activity was investigated in the wild-type strain as well as the Δ*murR* mutant. The results indicated that *murR* is a negative regulator since its deletion resulted in the stronger P_*murQ*_ activity (Fig. 8B). The P_*ptsA*_ activity remained unchanged in the presence or absence of *murR*, showing that the expression of *ptsA* is not regulated by the putative MurNAc utilization system (Fig. 8B). Finally, primer extension study was carried out in order to determine the transcription start site (TSS) of *ptsA*. The TSS of *ptsA* was a T located 47 bp upstream of the start codon of *ptsA* (Fig. 8C). The promoter core elements of *ptsA* consisted of TTGAAG, as the −35 box, and TGGTTAAAT, as an extended −10 box, which was an arrangement highly similar to that of the housekeeping promoters recognized by σ^A^ (Fig. 8D).

## DISCUSSION

Bacillus subtilis contains 16 known PTS transporters for the uptake of carbohydrates; among the 16, 6 transporters, i.e., MalP, MurP, NagP, SacP, SacX and TreP, lack the EIIA domain. There were three different possibilities for the phosphorylation of these EIIA-deficient PTS transporters: (i) specific phosphorylation of their EIIB domains by the two unknown cytoplasmic EIIAs, namely, *ptsA* (formerly *ypqE*) and *yyzE*; (ii) direct phosphorylation of the EIIB domains by HPr(H15∼P); and (iii) unspecific phosphorylation of the EIIB domains by the noncognate EIIA domains of other transporters. Single deletion of the EIIA domains indicated cross talk among the PTS transporters, removing the possibility of the presence of a specific EIIA for these EIIA-deficient PTS transporters. On the other hand, the ΔEIIA strain (KM455) was unable to grow (or grew weakly) with maltose, GlcNAc, sucrose, or trehalose. This result removed the possibility of direct phosphorylation of EIIA-deficient PTS transporters by HPr(H15∼P). It seems likely that the EIIA-deficient PTS transporters are phosphorylated by the EIIA domains of the glucose family of PTS transporters, namely, PtsG, GamP, and PtsA (formerly YpqE) in B. subtilis.

The glucose family of PTS transporters consisting of two glucose and glucoside subfamilies is the most highly represented family of transporters among 77 bacterial species (41). The cross talk among the EIIA and EIIB domains of this family is common. In Listeria monocytogenes EGD-e, EIICB^Tre^ (encoded by *L. monocytogenes 1255* [*lmo1255*]) is assumed to be phosphorylated by EIIA^Glc^ encoded by *lmo1017* (42). The GlcNAc and trehalose transporters also share the EIIA^Glc^ subunit in Vibrio cholerae (43). In Borrelia burgdorferi, EIIA^Glc^ encoded by the *crr* gene probably phosphorylates EIICBs encoded by *ptsG*, *malX1*, and *malX2* (44). In E. coli, transporters supported by EIIA^Glc^ (encoded by *crr*) are AscF, PtsG, TreB, and probably MalX and GlvB (45, 46). Besides, only the triple mutant (*crr*, *man*-162, and *nagE*) of E. coli K-12 is unable to take up methyl α-glucosides or grow with glucose (47). Also, BglF (EIIBCA^Bgl^) is able to substitute EIIA^Glc^ for phosphorylation of PtsG (EIICB^Glc^) and ScrA in E. coli (48). After deletion of the *crr* gene in Salmonella enterica serovar Typhimurium, the uptake of methyl α-glucosides is reduced to 10% to 20% of the level seen with the wild-type strain. Hence, it was proposed that a membrane-bound EIIA^Glc^-like protein substitutes for the cytoplasmic EIIA^Glc^ (49). Phylogenetic analysis within the glucose family of PTS transporters in E. coli revealed that the B. subtilis glucose family of PTS transporters clusters with their E. coli orthologues. Likewise, analysis of the EIIA domains indicated that EIIA^Glc^ is distinct from other EIIA families. This explains why EIIA^Glc^ is highly flexible for different EIIB domains, resulting in cross talk between EIIA and EIIB, which belong to the same family of PTS transporters (45, 50). Our results in this study also support the idea of the flexibility of the EIIAs of the glucose family of PTS transporters in B. subtilis.

The EIIA domain in the glucose family of PTS transporters is either membrane bound, as in the case of B. subtilis PtsG, or cytoplasmic, as in the case of E. coli EIIA^Glc^ (Crr) or B. subtilis PtsA. Structurally, the EIIA^Glc^ protein in E. coli has an unstructured N-terminal tail bound to a globular core which is made by an antiparallel β-sheet sandwich (51). This N-terminal tail is essential for inhibition of lactose and maltose transporters in *S*. Typhimurium or for inhibition of the ATPase activity of MalK in E. coli (51–55). It is suggested that the N terminus of EIIA^Glc^ is capable of binding to a negatively charged E. coli membrane surface. A two-state conformation was proposed for E. coli EIIA^Glc^. In the cytosol, a disordered N terminus is connected to the globular core, while the N terminus forms a helical conformation during the interaction between the EIIA globular core and the EIIB domain of EIICB^Glc^. Apparently, removal of the EIIA^Glc^ N terminus (EIIA^Glc^_fast_) (54) in *S*. Typhimurium makes it a very poor membrane anchor although it does not affect its phosphoryl acceptance from the HPr(H15∼P) (51). After dissection of the PtsG domains in our study, only the membrane-bound EIIA^Glc^ domain supported the growth of the cells with glucose, whereas their cytoplasmic versions were defective. The lack of properly folded proteins cannot be ruled out as a reason for these negative results. However, the absence of the N-terminal domain in the truncated version of B. subtilis EIIA^Glc^ might also be the reason. Bioinformatic analysis of PtsG showed that there is a helix secondary structure next to the C terminus of EIIB domain within the spacer sequence of EIIB-EIIA domains in EIIBCA^Glc^ (see Fig. S9 in the supplemental material). Nevertheless, the presence of this linker did not support the growth of the cells with glucose in strain KM380 (Fig. 4). Therefore, the domain linkers in the latter constructs were truncated in the middle of the sequence for fusion to TkmA. In contrast to EIIA^Glc^, the PtsA protein was functional regardless of its localization (cytoplasmic or membrane bound). This could have been due to the presence of 35 amino acids in the N terminus of PtsA and to its conformation causing this different mode of activity between PtsA and EIIA^Glc^. It must be noted that PtsA might also have functions other than the transfer of the phosphoryl group in the PTS as reported for E. coli EIIA^Glc^.

Surprisingly, both the TkmA-EIIA^Glc^ and TkmA-EIIBA^Glc^ fusions were able to phosphorylate EIIB^Mal^, EIIB^Nag^, EIIB^Sac^, and EIIB^Tre^. This was in contrast to the results obtained in the medium with glucose (Fig. 5B). The PTS transporters apparently form homodimers in order to transport the sugars (56). In E. coli, it was shown that phosphoryl transfer between EIIB and EIIA on IICB^wt^A^H554A^::EIICB^C384H^A^wt^ heterodimers is efficient. In contrast, phosphoryl transfer between EIIB and the sugar bound to EIIC, for instance, in EIIC^H195A^B^wt^A::EIIC^wt^B^C384H^A, was less efficient (50). This was due to the almost doubled distance of the cysteine in the active site of EIIB from the sugar binding pocket of EIIC, which is 30 Å between the sites of the same subunit whereas it is 70 Å between the sites of different subunits. Likewise, the linker between EIIC and the EIIB domain of PtsG in E. coli contains the KTPGRED motif, whose deletion or mutation is more deleterious for transport and phosphorylation than its absence (57). Expression of EIIC and EIIB split within their linker results in the retention of complete transport and phosphotransfer activity (58). Therefore, it seems that separation of the EIIB domain from the EIIC domain results in inefficient phosphorylation of glucose by PtsG if we exclude the possibility of protein stability or misfolding (see KM920 data in Fig. 5). Nevertheless, it seems that the presence of the EIIB domain (probably in its phosphorylated form) adjacent to EIIA stimulates its phosphoryl transfer to the noncognate EIIA-deficient PTS transporters, such as MalP.

In conclusion, we were able to show that the EIIAs of the glucose family of PTS transporters can compensate for the absence of other EIIAs in this family and phosphorylate the EIIA-deficient PTS transporters in B. subtilis.

## MATERIALS AND METHODS

### Strains, media, and growth conditions.

Bacterial strains used in this study are listed in Table S1 in the supplemental material. Escherichia coli JM109 was used for plasmid propagation (59) and JW2409-1 (Δ*ptsI*) for protein expression (40). Unless otherwise specified, Bacillus subtilis KM0, the tryptophan prototroph derivative of strain 168, was exploited as the wild-type strain (60). Transformants of E. coli were selected on LB agar (61) supplemented with ampicillin (100 μg/ml) or spectinomycin (100 μg/ml) depending on the plasmid antibiotic marker. Transformants of B. subtilis with a plasmid or gene deletion or integration were selected on LB agar containing spectinomycin (100 μg/ml), chloramphenicol (5 μg/ml), or erythromycin (5 μg/ml). Histidine prototroph transformants were cultured on Spizizen's minimal medium [(NH_4_)_2_SO_4_ (2 g/liter), K_2_HPO_4_ (14 g/liter), KH_2_PO_4_ (6 g/liter), Na_3_ citrate · 2H_2_O (1 g/liter), MgSO_4_ · 7H_2_O (0.2 g/liter)] (62), while the histidine auxotroph mutants were selected on Spizizen's minimal medium with 20 μg/ml l-histidine. The tryptophan auxotroph mutants were cultured on minimal medium supplemented with 50 μg/ml l-tryptophan. For transformation of B. subtilis, Casamino Acids were added to the Spizizen's minimal salt at final concentrations of 0.02% (wt/vol) (medium I) and 0.01% (wt/vol) (medium II) for the transformation media. Unless otherwise specified, glucose or glucitol with a final concentration of 0.5% was added to all minimal media as the main carbon source.

The growth of B. subtilis strains with different carbon sources was investigated in Spizizen's minimal salt medium without sodium citrate. A 600-μl volume of trace element solution (CaCl_2_ · 2H_2_O [0.5 g/liter], FeCl_3_ · 6H_2_O [16.7 g/liter], Na_2_ EDTA [20.1 g/liter], ZnSO_4_ · 7H_2_O [0.18 g/liter], MnSO_4_ · H_2_O [0.1 g/liter], CuSO_4_ · 5H_2_O [0.16 g/liter], CoCl_2_ · 6H_2_O [0.18 g/liter]) was added to 200 ml of the Spizizen's minimal salt medium. The growth medium was also supplemented with glutamate (20 μg/ml) and Kao and Michayluk vitamin solution (catalog no. K3129; Sigma-Aldrich, USA). As a sole carbon source, glucitol was added to the overnight culture, whereas GlcNAc, maltose, sucrose, or trehalose was added to the main cultures with a final concentration of 0.5% (wt/vol). Each of the 5-ml overnight cultures was centrifuged, and the cell pellets were washed once and resuspended in Spizizen's minimal salt medium. Unless otherwise specified, the growth of the strains was measured in 24-well microtiter plates (2-ml capacity) using a Spark microplate reader (Tecan, Männedorf, Switzerland). Each main culture with 1 ml minimal medium containing 0.5% of the desired carbohydrate was inoculated with a starting OD_600_ of 0.01. It should be noted that the starting OD_600_, measured at 0.01 by an Ultrospec 3000 UV-visible light (UV-Vis) spectrophotometer (Pharmacia Biotech), was measured at approximately 0.05 by the Spark microplate reader. The growth of the strains was then monitored at 4-h intervals up to 24 h, and the average growth rate was calculated. For experiments performed using 100-ml Erlenmeyer flasks, 8 ml of culture medium was inoculated with a starting OD_600_ of 0.1 and the growth of the cells was measured after 0 h, 6 h, and 24 h of inoculation. For overexpression of the truncated *ptsG* variants in B. subtilis, 1 mM IPTG was added upon inoculation into the overnight and main cultures for induction of P_*hyperspank*_. In the latter case, the uninduced main cultures were inoculated from uninduced overnight cultures.

To induce the bacterial cells carrying the *lacZ* reporter gene, 80 ml LB medium with spectinomycin (100 μg/ml) was inoculated with a starting OD_600_ of 0.05. After 2 h of incubation at 37°C and 200 rpm shaking, 0.2% of each carbohydrate, including arbutin (Sigma-Aldrich, USA), fructose (Merck, Germany), glucose (Merck, Germany), locust bean gum from Ceratonia (Sigma-Aldrich, USA), mannitol (Carl Roth, Germany), GlcNAc (Fluka, Switzerland), trehalose (Carl Roth, Germany), cellobiose (Sigma-Aldrich, USA), glucosamine (Fluka, Switzerland), glycerol (Sigma-Aldrich, USA), maltose (Sigma-Aldrich, USA), mannose (Amresco, USA), and sucrose (Carl Roth, Germany), was individually added to the 5-ml aliquots of the bacterial cultures. The cells were harvested 1 h after the addition of carbohydrates and used for the β-galactosidase assay. All experiments were performed at least 3 times, and the mean values were used for the analysis.

Overexpression of the desired proteins was carried out in E. coli strain JW2409-1 harboring plasmid pMW850.2, pMW851.2, or pKAM401 carrying an l-rhamnose inducible promoter, *rha*P_*BAD*_. A single colony of E. coli was inoculated into 5-ml LB with ampicillin, and the culture was incubated overnight. The overnight culture was used for inoculation of 100 ml LB medium with ampicillin (main culture) with a starting OD_600_ of 0.5. The main culture was primarily incubated for 2 h at 37°C followed by addition of 0.2% l-rhamnose as the inducer. Afterwards, the culture was incubated at 37°C and harvested after 6 h of cultivation. The cell pellet was kept at −20°C prior to protein purification.

### DNA manipulation and plasmid construction.

Standard molecular techniques were performed according to the work of Sambrook et al. (63). Plasmids constructed in this study are listed in Table S2. To amplify the desired DNA fragment, PCR was performed using Phusion Hot Start II high-fidelity DNA polymerase from Fisher Scientific GmbH (Schwerte, Germany) on a PTC-200 Peltier thermal cycler (MJ Research Inc.). DNA fragments were fused in a fusion PCR or by Gibson assembly (catalog no. E2611L; New England BioLabs, Frankfurt am Main, Germany). Genomic DNA was isolated from B. subtilis strains by the use of a DNeasy blood and tissue kit (catalog no. 69506; Qiagen, Hilden, Germany). The oligonucleotides were synthesized by Eurofins MWG Operons (Ebersberg, Germany) (Table S3). Restriction enzymes were purchased from New England BioLabs (Frankfurt am Main, Germany). T4 DNA ligase was provided by Thermo Fisher Scientific Inc. (St. Leon-Rot, Germany). Digested DNA fragments from agarose gel and amplified DNAs in PCRs were extracted using a NucleoSpin gel and PCR cleanup kit (Macherey-Nagel, Düren, Germany). Plasmid DNA was extracted from E. coli using an innuPREP plasmid mini kit (Analytic Jena AG, Jena, Germany). DNA constructs were sequenced by GATC Biotech AG (Konstanz, Germany). Transformation of E. coli strains JM109 and JW2409-1 was carried out as described before (64).

### Construction of B. subtilis strains.

Transformation of B. subtilis strains was carried out by a natural transformation protocol (Paris method) (65). Different strategies were used for markerless deletion of the target genes from the chromosome of B. subtilis. Unless otherwise specified, the start and stop codons of the target genes were fused in order to prevent the polar effect in all deletion strategies. In the first strategy, desired genes were deleted using temperature-sensitive derivatives of the pMW521.1 plasmid (66). pMW521.1 is an integration shuttle vector consisting of *ori*_pUC18_ for E. coli and temperature-sensitive *ori*_pE194_ for B. subtilis. The deletion cassette was constructed by amplification of the upstream and downstream flanking regions of the target gene followed by fusion PCRs or insertion of the DNA fragments via ligation of 3 fragments into pMW521.1 as described in Table S2. After transformation of the host strain, the cells with the integrated pMW521.1 derivatives resulting from a single crossover in their chromosome were selected at 50°C on LB-containing spectinomycin plates. Afterwards, a single colony was inoculated into LB without an antibiotic and cultured for 24 h at 30°C. The 10^−6^ dilution of the bacterial culture was then plated on LB without an antibiotic and incubated at 50°C. Finally, single colonies were tested for loss of spectinomycin resistance and deletion of the target gene.

The second strategy was based on the site-specific *mroxP*-Cre recombination system developed by L. Warth and J. Altenbuchner (67). The derivatives of plasmid pKAM19 (68) carrying the upstream flanking-*mroxP-cat-mroxP*-downstream flanking cassette were transferred into the target B. subtilis cells. Transformants were first selected on LB containing chloramphenicol. Afterwards, the chloramphenicol-resistant cells were transformed with unstable plasmid pJOE6732.1 expressing the Cre recombinase and selected on LB with spectinomycin. Afterwards, a single colony was further cultured in LB for 24 h at 37°C and a 10^−6^ dilution was plated on LB without an antibiotic. The colonies were checked for the loss of spectinomycin and chloramphenicol and for deletion of the target genes. To delete some of genes, genomic DNA of the BKE (*B**acillus* knockout erythromycin) strains carrying the deletion of the target gene was used (69). In this case, the erythromycin resistance gene was then removed using pJOE6732.1 in the same manner.

In the third strategy, the desired gene was deleted using the derivatives of pJOE6743.1, namely, pJOE7644.2 and pJOE8525, based on the mannose markerless-deletion system developed by M. Wenzel and J. Altenbuchner (70). In this system, strain KM296 (Δ*manPA*::*ermC*) was transformed and the transformants were cultured on LB containing spectinomycin to select the transformants harboring the integrated plasmid via single crossover. Next, a single colony was inoculated into 1 ml LB medium and cultured for 4 h. Subsequently, a 10^−3^ dilution of the cells was inoculated into 1 ml LB medium with 0.5% mannose. After 4 h of cultivation, a 10^−6^ dilution of the bacterial culture was plated on LB plates with 0.5% mannose. Finally, the single colonies were tested for the loss of spectinomycin resistance gene and deletion of the target gene.

In the last strategy, markerless gene deletion was carried out based on the CRISPR/Cas9 system developed by J. Altenbuchner (71). Plasmid pJOE8999 carrying the *cas9* gene under the control of B. subtilis mannose-inducible promoter (P_*manP*_) and the genomic RNA (gRNA) gene sequence under the control of Corynebacterium glutamicum P_*vanA*_ was used as the parental plasmid for construction of pKAM412. The procedure of the selection of correct transformants has been explained thoroughly before (71). For markerless integration of the genes, the pHM30/pHM31 system based on histidine auxo- and prototrophy was used (72). Complementation with PTS transporters in strain KM455 was carried out by amplification of their encoding genes from KM0 (wild type) in PCRs. To improve the efficiency of the homologous recombination, the up- and downstream flanking regions of genes were longer than 1,000 bp (Table S1).

### Primer extension.

To identify the transcription start site (TSS) of *ypqE*, strain KM455 was transformed with pKAM299, which contained *ori*_pUB110_ and carried the P_*ypqE*_-*eGFP* fusion. Strain KM455 pKAM299 was inoculated into 10 ml LB_spc_, and the bacterial culture was incubated overnight at 37°C. After centrifugation of about 2 × 10^9^ of KM455 pKAM299 cells, the total mRNA was isolated from the cell pellet by the use of a Qiagen RNeasy minikit (Hilden, Germany) according to the manufacturer's instruction. Approximately 65 μg of total RNA was precipitated using sodium acetate (3 M, pH 6.3) and ethanol. The RNA pellet was then dissolved in 4 μl of RNase-free double-distilled water (ddH_2_O) and incubated at 65°C for 3 min. Next, 0.5 μl of Cy5-labeled s8484 oligonucleotide (10 pmol/μl), 0.5 μl of murine RNase inhibitor (New England BioLabs GmbH, Frankfurt am Main, Germany), 2 μl of ProtoScript II reverse transcriptase buffer (New England BioLabs GmbH, Frankfurt am Main, Germany) (5×), and 1 μl dithiothreitol (DTT) (0.1 M) were added. The mixture was then incubated for 20 min at 56°C followed by incubation for 5 min at room temperature (RT). To start the reverse transcription, 1 μl deoxynucleoside triphosphate (dNTP) (10 mM) and 1 μl ProtoScript II reverse transcriptase (New England BioLabs GmbH, Frankfurt am Main, Germany) (200 U/μl) were added and the reaction was incubated for 1 h at 42°C. Finally, the generated cDNA was purified using a DNA Clean & Concentrator-5 kit (Zymo Research GmbH, Freiburg, Germany) and eluted in 6 μl ddH_2_O. To run the sample on a CEQ 8000 DNA analysis system (Beckman Coulter Inc., Brea, CA), 34 μl of sample loading solution (GenomeLab; Beckman Coulter Inc., Brea, CA) and 0.5 μl of DNA Size Standard Kit 600 (GenomeLab; Beckman Coulter Inc., Brea, CA) were added. The result of the reverse transcription reaction was then compared with the sequencing results of the pKAM299 analysis using s8484 oligonucleotide and CEQ 8000 DNA Analysis System software (Beckman Coulter Inc., Brea, CA).

### DNA sequencing.

The sequencing of pKAM299 with the s8484 oligonucleotide was performed using a Thermo Sequenase cycle sequencing kit (Affymetrix, High Wycombe, United Kingdom). The sequencing master mix was prepared by mixing 2 μl of pKAM299 (30 fmol/μl) with 2 μl of the reaction buffer, 1 μl of the s8484 oligonucleotide (4 pmol/μl), 1 μl dimethyl sulfoxide (DMSO), 2 μl DNA polymerase, and 9.5 μl ddH_2_O. A 4-μl volume of the sequencing master mix was then added to 4-μl aliquots of ddGTP, ddATP, ddTTP, and ddCTP. Using a PTC-200 Peltier thermal cycler (MJ Research Inc., Watertown, MA), the sequencing reaction was accomplished. The amplification program was as follows: initial denaturation for 2 min at 95°C; 30 cycles of 95°C for 30 s, 56°C for 30 s, and 72°C for 1 min; and final extension for 1 min at 72°C. Finally, amplified DNA in each reaction was precipitated with sodium acetate (3 M, pH 6.3) and ethanol. The DNA was then dissolved in 40 μl sample loading solution (GenomeLab; Beckman Coulter Inc., Brea, CA). After addition of 0.5 μl DNA Size Standard Kit 600 (GenomeLab; Beckman Coulter Inc., Brea, CA), the samples were run on a CEQ 8000 DNA analysis system (Beckman Coulter Inc., Brea, CA) and the results were analyzed using CEQ 8000 DNA Analysis System software (Beckman Coulter Inc., Brea, CA).

### Protein purification.

To purify the desired proteins for the *in vitro* phosphorylation experiment, a cell pellet of E. coli strain JW2409-1 carrying expression plasmid pMW850.2, pMW851.2, or pKAM401 was resuspended in 10 ml of resuspension buffer containing 0.1 M Tris-HCl (pH 7.8), 0.3 NaCl, and 1 mM tris(2-carboxyethyl)phosphine (TCEP). After disruption of the cells by the use of a EmulsiFlex-C5 high-pressure homogenizer (Avestin, Mannheim, Germany) at 15,000 lb/in^2^, the crude extract was centrifuged for 30 min at 12,000 × *g*. The cleared lysate was then passed through 1 ml of Talon metal affinity resin (Clontech Laboratories, Inc., Mountain View, CA) for His_6_-tagged proteins or Strep-Tactin resin for purification of streptavidin (Strep)-tagged proteins. The purification steps were carried out according to the manufacturer's instructions. After the resins were washed with the resuspension buffer, 150 mM imidazole (His_6_-tagged proteins) or 2.5 mM desthiobiotin (Strep-tagged proteins) was added to the resuspension buffer for elution of the proteins. Finally, the protein concentration was determined as described by Bradford (73) with bovine serum albumin as a standard.

### *In vitro* phosphorylation of proteins.

To phosphorylate the purified components of the PTS cascade *in vitro*, [^32^P]phosphoenolpyruvate was synthesized in a reaction using pyruvate kinase from rabbit muscle (catalog no. 000000010128155001; Sigma-Aldrich) (∼200 units/mg) and [γ-^32^P]ATP (PerkinElmer) (3,000 Ci/mmol, 10 mCi/ml) according to the method described by Roossien et al. (74) with the following modifications. Each phosphorylation reaction was prepared in a total volume of 19 μl by mixing 2 μl pyruvate kinase, 2 μl of the 10× reaction buffer (0.5 M HEPES [pH 7.4], 50 mM MgCl_2_, 150 mM KCl, 25 μM sodium pyruvate, 25 nM PEP) with or without 1 μl enzyme I (0.7 μg/μl), 1 μl HPr (0.8 μg/μl), 10 μl YpqE (0.15 μg/μl) and ddH_2_O. A 1-μl volume of [γ-^32^P]ATP was added to each reaction mixture, and the reaction mixtures were incubated for 3 h at 37°C. Each reaction was then stopped by adding SDS-PAGE sample buffer and denaturation of the proteins for 10 min at 100°C. The proteins were subsequently separated on a 15% SDS-PAGE gel. After exposure of the gels to a phosphor screen overnight, the results were visualized by the use of a phosphorimager (Storm 860 phosphorimager; Molecular Dynamics).

### Measurement of β-galactosidase activity.

The levels of β-galactosidase activity of the strains were measured with *o*-nitrophenyl-β-galactopyranoside (oNPG) according to the method described by Miller (75) and the modification described by Wenzel and Altenbuchner (76). Since the presence of arbutin in the culture medium interfered with the β-galactosidase due to formation of the red coloring, the cells were once washed with LB and the pellet was dissolved in 1 ml of buffer Z.

## Supplementary Material

Supplemental file 1
