# Supplementary material for: Cross Talk among Transporters of the Phosphoenolpyruvate-Dependent Phosphotransferase System in Bacillus subtilis
Source: J Bacteriol. 2018 Sep 10;200(19):e00213-18. doi: 10.1128/JB.00213-18 (PMC6148471; doi:10.1128/JB.00213-18)
Supplement: Supplemental file 1 [file zjb019184879s1.pdf]

Supplementary materials

Cross-talk among transporters of the phosphoenolpyruvate-dependent phosphotransferase system in *Bacillus subtilis*

Kambiz Morabbi Heravi* and Josef Altenbuchner

Institut für Industrielle Genetik, Universität Stuttgart, Allmandring 31, 70569 Stuttgart, Germany

Running title: Cross-talk among PTS transporters

Keywords: carbohydrate uptake, phosphotransfer, enzyme II, permease

* **For correspondence:**

E-mail: kambiz.morabbi@iig.uni-stuttgart.de

Tel.: +49 711 685 66972

Fax: +49 711 685 66973

Materials and Methods

TABLE S1 Strains used in this study.

Strain or plasmid	Genotype or relevant structure	Source, reference or description
E. coli		
JM109	*recA1*, *endA1*, *gyrA96*, *thi*-1, *hsdR17*(r_K^-, m_k^+), *supE44*, *gyrA96*, *relA1*, λ^-, Δ(*lac-proAB*), F'(*traD*36, *proAB*$^+$, *lacI*^{q}, (Δ*lacZ*)M15)	(1)
JW2409-1	Δ(*araD-araB*)567, Δ*lacZ*4787(::*rrnB*-3), λ^-, Δ*ptsI*745::*kan*, *rph-1*, Δ(*rhaD-rhaB*)568, *hsdR*514	(2)
B. subtilis		
BKE01690	*trpC2* Δ*murR*::*loxP-ermC-loxP*	(3)
BKE05820	*trpC2* Δ*gmuA*::*loxP-ermC-loxP*	(3)
BKE07700	*trpC2* Δ*nagP*::*loxP-ermC-loxP*	(3)
BKE08200	*trpC2* Δ*malP*::*loxP-ermC-loxP*	(3)
BKE12010	*trpC2* Δ*manP*::*loxP-ermC-loxP*	(3)
BKE14400	*trpC2* Δ*fruA*::*loxP-ermC-loxP*	(3)
BKE24850	*trpC2* Δ*glcK*::*loxP-ermC-loxP*	(3)
BKE38050	*trpC2* Δ*sacP*::*loxP-ermC-loxP*	(3)
BKE38570	*trpC2* Δ*licA*::*loxP-ermC-loxP*	(3)
BKE38580	*trpC2* Δ*licC*::*loxP-ermC-loxP*	(3)
BKE39270	*trpC2* Δ*bglP*::*loxP-ermC-loxP*	(3)
KM0	strain 168 *trp*$^+$	(4)
KM272	Δ*ypqE*	pKAM224 → KM0
KM280	Δ*ptsG* Δ*glcK*::*loxP-ermC-loxP*	gDNA[2] BKE24850 → KM364
KM281	Δ*ptsG* Δ*glcK*::*loxP*	pJOE6732.1 → KM280
KM285	Δ*licBCAH*	pKAM239 → KM0

KM287	Δ*levDEFG*	pKAM234 → KM0
KM288	Δ*yyzE*	pKAM235 → KM0
KM290	Δ*ypqE* Δ*yyzE*	pKAM235 → KM272
KM291	Δ*ypqE* Δ*yyzE* Δ*levDEFG*	pKAM234 → KM290
KM292	Δ*ypqE* Δ*yyzE* Δ*levDEFG* Δ*mtlF*::*mroxP-cat-mroxP*	pKAM47 → KM291
KM293	Δ*ypqE* Δ*yyzE* Δ*levDEFG* Δ*mtlF*::*mrmrP*	pJOE6732.1 → KM292
KM294	Δ*ypqE* Δ*yyzE* Δ*levDEFG* Δ*mtlF*::*mrmrP* Δ*mdxRDEFGyvdJmalKLpgcM*	pKAM238 → KM293
KM295	Δ*ypqE* Δ*yyzE* Δ*levDEFG* Δ*mtlF*::*mrmrP* Δ*mdxRDEFGyvdJmalKLpgcM* Δ[*ptsG*-P_{ptsHI}]::*ermC*	pMW312.2 → KM294
KM296	Δ*manPA*::*ermC*	pJOE6577.1 → KM0
KM297	Δ*manPA*	pJOE7644.2 → KM296
KM320	Δ*ypqE* Δ*yyzE* Δ*levDEFG* Δ*mtlF*::*mrmrP* Δ*mdxRDEFGyvdJmalKLpgcM* Δ*ptsG*	pMW373.3 → KM295
KM326	Δ*ypqE* Δ*yyzE* Δ*levDEFG* Δ*mtlF*::*mrmrP* Δ*mdxRDEFGyvdJmalKLpgcM* Δ*ptsG* Δ*gamP*::*mroxP-cat-mroxP*	pKAM286 → KM320
KM337	Δ*manPA* Δ*treP*::*mroxP-cat-mroxP*	pKAM269 → KM297
KM338	Δ*manPA* Δ*treP*::*mrmrP*	pJOE6732.1 → KM337
KM357	Δ*mtlF*::*mroxP-cat-mroxP*	pKAM47 → KM0
KM358	Δ*mtlF*::*mrmrP*	pJOE6732.1 → KM357
KM359	Δ*manPA* Δ*mdxRDEFGyvdJmalKLpgcM*	pJOE8525.2 → KM297
KM363	Δ[*ptsG-ptsH′*]::*ermC*	pMW312.2 → KM0
KM364	Δ*ptsG*	pMW373.3 → KM363
KM365	Δ*gamP*::*mroxP-cat-mroxP*	pKAM286 → KM0
KM366	Δ*gamP*::*mrmrP*	pJOE6732.1 → KM365
KM369	Δ*gmuA*::*loxP-ermC-loxP*	pKAM041 → BKE05820
KM370	Δ*glcK*::*loxP-ermC-loxP*	pKAM041 → BKE24850
KM373	Δ*gmuA*::*loxP*	pJOE6732.1 → KM369

KM374	Δ*glcK*::*loxP*	pJOE6732.1 → KM370
KM378	Δ*manPA* Δ*mdxRDEFGyvdJmalKLpgcM* Δ*malP*::*loxP-ermC-loxP*	gDNA BKE08200 → KM359
KM379	Δ*glcK*::*loxP* Δ[*ptsG*-P_{ptsHI}-*ptsH*]::*ermC*	pKAM172 → KM374
KM380	Δ*glcK*::*loxP* *ptsG*(EIICB-linkerEIIA)	pKAM173 → KM379
KM381	Δ*glcK*::*loxP* *ptsG*(EIICBlinker-EIIA)	pKAM174 → KM379
KM382	Δ*glcK*::*loxP* *ptsG*(EIICB-EIIA)	pKAM175 → KM379
KM402	Δ*manPA* Δ*mdxRDEFGyvdJmalKLpgcM* Δ*malP*::*loxP*	pJOE6732.1 → KM378
KM410	Δ*nagP*::*loxP-ermC-loxP*	pKAM041 → BKE07700
KM414	Δ*sacP*::*loxP-ermC-loxP*	pKAM041 → BKE38050
KM415	Δ*bglP*::*loxP-ermC-loxP*	pKAM041 → BKE39270
KM418	Δ*nagP*::*loxP*	pJOE6732.1 → KM410
KM422	Δ*sacP*::*loxP*	pJOE6732.1 → KM414
KM423	Δ*bglP*::*loxP*	pJOE6732.1 → KM415
KM432	Δ*fruA*::*loxP-ermC-loxP*	pKAM041 → BKE14400
KM435	Δ*fruA*::*loxP*	pJOE6732.1 → KM432
KM440	Δ*ypqE* Δ*yyzE* Δ*levDEFG* Δ*mtlF*::*mrmrP* Δ*mdxRDEFGyvdJmalKLpgcM* Δ*ptsG* Δ*gamP*::*mrmrP*	pJOE6732.1 → KM326
KM441	Δ*ypqE* Δ*yyzE* Δ*levDEFG* Δ*mtlF*::*mrmrP* Δ*mdxRDEFGyvdJmalKLpgcM* Δ*ptsG* Δ*gamP*::*mrmrP* Δ*bglP*::*loxP-ermC-loxP*	gDNA BKE39270 → KM440
KM442	Δ*ypqE* Δ*yyzE* Δ*levDEFG* Δ*mtlF*::*mrmrP* Δ*mdxRDEFGyvdJmalKLpgcM* Δ*ptsG* Δ*gamP*::*mrmrP* Δ*bglP*::*loxP*	pJOE6732.1 → KM441
KM443	Δ*ypqE* Δ*yyzE* Δ*levDEFG* Δ*mtlF*::*mrmrP* Δ*mdxRDEFGyvdJmalKLpgcM* Δ*ptsG* Δ*gamP*::*mrmrP* Δ*bglP*::*loxP* Δ*licA*::*loxP-ermC-loxP*	gDNA BKE38570 → KM442
KM444	Δ*ypqE* Δ*yyzE* Δ*levDEFG* Δ*mtlF*::*mrmrP* Δ*mdxRDEFGyvdJmalKLpgcM* Δ*ptsG* Δ*gamP*::*mrmrP* Δ*bglP*::*loxP* Δ*licA*::*loxP*	pJOE6732.1 → KM443
KM445	Δ*glcK*::*loxP* *'ptsG*(EIICB, ΔEIIA)	pKAM306 → KM379

KM446	Δ*glcK*::*loxP* '*ptsG*(EIIC, ΔEIIBA)	pKAM307 → KM379
KM447	Δ*glcK*::*loxP* '*ptsG*(EIIC-EIIA, ΔEIIB)	pKAM308 → KM379
KM448	Δ*glcK*::*loxP* '*ptsG*(EIIC-EIIBA)	Primary PCRs<br>s9923-s9828 and s9829-s5624<br>(Gibson assembly)<br>Final PCR s7582-s7711 → KM379
KM449	Δ*glcK*::*loxP* '*ptsG*(EIIA, ΔEIICB)	Primary PCRs<br>s5621-s9831 and s9832-s5624<br>(Gibson assembly)<br>Final PCR s9830-s7711 → KM379
KM450	Δ*ypqE* Δ*yyzE* Δ*levDEFG* Δ*mtlF*::*mrmrP* Δ*mdxRDEFGyvdJmalKLpgcM* Δ*ptsG* Δ*gamP*::*mrmrP* Δ*bglP*::*loxP* Δ*licA*::*loxP* Δ*gmuA*::*loxP-ermC-loxP*	gDNA BKE05820 → KM444
KM451	Δ*ypqE* Δ*yyzE* Δ*levDEFG* Δ*mtlF*::*mrmrP* Δ*mdxRDEFGyvdJmalKLpgcM* Δ*ptsG* Δ*gamP*::*mrmrP* Δ*bglP*::*loxP* Δ*licA*::*loxP* Δ*gmuA*::*loxP*	pJOE6732.1 → KM450
KM452	Δ*ypqE* Δ*yyzE* Δ*levDEFG* Δ*mtlF*::*mrmrP* Δ*mdxRDEFGyvdJmalKLpgcM* Δ*ptsG* Δ*gamP*::*mrmrP* Δ*bglP*::*loxP* Δ*licA*::*loxP* Δ*gmuA*::*loxP* Δ*manP*::*loxP-ermC-loxP*	gDNA BKE12010 → KM451
KM453	Δ*ypqE* Δ*yyzE* Δ*levDEFG* Δ*mtlF*::*mrmrP* Δ*mdxRDEFGyvdJmalKLpgcM* Δ*ptsG* Δ*gamP*::*mrmrP* Δ*bglP*::*loxP* Δ*licA*::*loxP* Δ*gmuA*::*loxP* Δ*manP*::*loxP*	pJOE6732.1 → KM452
KM454	Δ*ypqE* Δ*yyzE* Δ*levDEFG* Δ*mtlF*::*mrmrP* Δ*mdxRDEFGyvdJmalKLpgcM* Δ*ptsG* Δ*gamP*::*mrmrP* Δ*bglP*::*loxP* Δ*licA*::*loxP* Δ*gmuA*::*loxP* Δ*manP*::*loxP* Δ*fruA*::*loxP-ermC-loxP*	gDNA BKE14400 → KM453
KM455	Δ*ypqE* Δ*yyzE* Δ*levDEFG* Δ*mtlF*::*mrmrP* Δ*mdxRDEFGyvdJmalKLpgcM* Δ*ptsG* Δ*gamP*::*mrmrP* Δ*bglP*::*loxP* Δ*licA*::*loxP* Δ*gmuA*::*loxP* Δ*manP*::*loxP* Δ*fruA*::*loxP*	pJOE6732.1 → KM454
KM645	Δ*manP*::*loxP-ermC-loxP*	pKAM041 → BKE12010
KM646	Δ*manP*::*loxP*	pJOE6732.1 → KM645
KM679	*amyE*::[*ter*-P_{ypqE}-*lacZ*, *spcR*]	pKAM292 → KM0

KM768	Δ*glcK*::*loxP ptsG*(EIIBA, ΔEIIC)	Primary PCRs<br>s5621-s10595 and s10596-s5624<br>(Gibson assembly)<br>Final PCR s9830-s7711 → KM379
KM790	*ptsG*$^+$ Δ*ypqE* Δ*yyzE* Δ*levDEFG* Δ*mtlF*::*mrmrP* Δ*mdxRDEFGyvdJmalKLpgcM* Δ*gamP*::*mrmrP* Δ*bglP*::*loxP* Δ*licA*::*loxP* Δ*gmuA*::*loxP* Δ*manP*::*loxP* Δ*fruA*::*loxP*	PCR s10727-s10728 → KM455
KM791	*levDEFG*$^+$ Δ*ypqE* Δ*yyzE* Δ*mtlF*::*mrmrP* Δ*mdxRDEFGyvdJmalKLpgcM* Δ*ptsG* Δ*gamP*::*mrmrP* Δ*bglP*::*loxP* Δ*licA*::*loxP* Δ*gmuA*::*loxP* Δ*manP*::*loxP* Δ*fruA*::*loxP*	PCR s8733-s8736 → KM455
KM792	*bglP*$^+$ Δ*ypqE* Δ*yyzE* Δ*levDEFG* Δ*mtlF*::*mrmrP* Δ*mdxRDEFGyvdJmalKLpgcM* Δ*ptsG* Δ*gamP*::*mrmrP* Δ*licA*::*loxP* Δ*gmuA*::*loxP* Δ*manP*::*loxP* Δ*fruA*::*loxP*	PCR s9888-s11429 → KM455
KM793	*licA*$^+$ Δ*ypqE* Δ*yyzE* Δ*levDEFG* Δ*mtlF*::*mrmrP* Δ*mdxRDEFGyvdJmalKLpgcM* Δ*ptsG* Δ*gamP*::*mrmrP* Δ*bglP*::*loxP* Δ*gmuA*::*loxP* Δ*manP*::*loxP* Δ*fruA*::*loxP*	PCR s9935-s9936 → KM455
KM794	*gamP*$^+$ Δ*ypqE* Δ*yyzE* Δ*levDEFG* Δ*mtlF*::*mrmrP* Δ*mdxRDEFGyvdJmalKLpgcM* Δ*ptsG* Δ*bglP*::*loxP* Δ*licA*::*loxP* Δ*gmuA*::*loxP* Δ*manP*::*loxP* Δ*fruA*::*loxP*	PCR s10860-s11430 → KM455
KM795	*mtlF*$^+$ Δ*ypqE* Δ*yyzE* Δ*levDEFG* Δ*mdxRDEFGyvdJmalKLpgcM* Δ*ptsG* Δ*gamP*::*mrmrP* Δ*bglP*::*loxP* Δ*licA*::*loxP* Δ*gmuA*::*loxP* Δ*manP*::*loxP* Δ*fruA*::*loxP*	PCR s6345-s6759 → KM455
KM796	*gmuA*$^+$ Δ*ypqE* Δ*yyzE* Δ*levDEFG* Δ*mtlF*::*mrmrP* Δ*mdxRDEFGyvdJmalKLpgcM* Δ*ptsG* Δ*gamP*::*mrmrP* Δ*bglP*::*loxP* Δ*licA*::*loxP* Δ*manP*::*loxP* Δ*fruA*::*loxP*	PCR s9994-s9995 → KM455
KM797	*manP*$^+$ Δ*ypqE* Δ*yyzE* Δ*levDEFG* Δ*mtlF*::*mrmrP* Δ*mdxRDEFGyvdJmalKLpgcM* Δ*ptsG* Δ*gamP*::*mrmrP* Δ*bglP*::*loxP* Δ*licA*::*loxP* Δ*gmuA*::*loxP* Δ*fruA*::*loxP*	PCR s8044-s8659 → KM455

KM800	*yvcA*'-*spcR*-'*hisI* Δ*ypqE* Δ*yyzE* Δ*levDEFG* Δ*mtlF*::*mrmrP* Δ*mdxRDEFGyvdJmalKLpgcM* Δ*ptsG* Δ*gamP*::*mrmrP* Δ*bglP*::*loxP* Δ*licA*::*loxP* Δ*gmuA*::*loxP* Δ*manP*::*loxP* Δ*fruA*::*loxP*	pHM30 → KM455
KM801	*yvcA*'-*ypqE*-P$_{ypqE}$-*hisI* Δ*ypqE* Δ*yyzE* Δ*levDEFG* Δ*mtlF*::*mrmrP* Δ*mdxRDEFGyvdJmalKLpgcM* Δ*ptsG* Δ*gamP*::*mrmrP* Δ*bglP*::*loxP* Δ*licA*::*loxP* Δ*gmuA*::*loxP* Δ*manP*::*loxP* Δ*fruA*::*loxP*	pKAM374 → KM800
KM802	*yvcA*'-*yyzE*-P$_{yyzE}$-*hisI* Δ*ypqE* Δ*yyzE* Δ*levDEFG* Δ*mtlF*::*mrmrP* Δ*mdxRDEFGyvdJmalKLpgcM* Δ*ptsG* Δ*gamP*::*mrmrP* Δ*bglP*::*loxP* Δ*licA*::*loxP* Δ*gmuA*::*loxP* Δ*manP*::*loxP* Δ*fruA*::*loxP*	pKAM375 → KM800
KM815	*yvcA*'-′*levR*-P$_{lev}$-*levD*-*hisI* Δ*ypqE* Δ*yyzE* Δ*levDEFG* Δ*mtlF*::*mrmrP* Δ*mdxRDEFGyvdJmalKLpgcM* Δ*ptsG* Δ*gamP*::*mrmrP* Δ*bglP*::*loxP* Δ*licA*::*loxP* Δ*gmuA*::*loxP* Δ*manP*::*loxP* Δ*fruA*::*loxP*	pKAM379 → KM800
KM819	Δ*licC*::*loxP*-*ermC*-*loxP* *licA*$^+$ Δ*ypqE* Δ*yyzE* Δ*levDEFG* Δ*mtlF*::*mrmrP* Δ*mdxRDEFGyvdJmalKLpgcM* Δ*ptsG* Δ*gamP*::*mrmrP* Δ*bglP*::*loxP* Δ*gmuA*::*loxP* Δ*manP*::*loxP* Δ*fruA*::*loxP*	PCR s11433-s11434 from BKE38580 → KM793
KM820	Δ*licC*::*loxP* *licA*$^+$ Δ*ypqE* Δ*yyzE* Δ*levDEFG* Δ*mtlF*::*mrmrP* Δ*mdxRDEFGyvdJmalKLpgcM* Δ*ptsG* Δ*gamP*::*mrmrP* Δ*bglP*::*loxP* Δ*gmuA*::*loxP* Δ*manP*::*loxP* Δ*fruA*::*loxP*	pJOE6732.1 → KM819
KM822	*amyE*::[*ter*-P$_{ypqE}$-*lacZ*, *spcR*] Δ*ypqE* Δ*yyzE* Δ*levDEFG* Δ*mtlF::mrmrP* Δ*mdxRDEFGyvdJmalKLpgcM* Δ*ptsG* Δ*gamP*::*mrmrP* Δ*bglP*::*loxP* Δ*licA*::*loxP* Δ*gmuA*::*loxP* Δ*manP*::*loxP* Δ*fruA*::*loxP*	pKAM292 → KM455
KM848	*trpC2* Δ*murR*::*loxP*	pJOE6732.1 → BKE01690
KM849	*trpC2* Δ*murR*::*loxP* *amyE*::[*ter*-P$_{ypqE}$-*lacZ*, *spcR*]	pKAM292 → KM848

KM870	*bglS*::[ter_{rrnB}-$P_{hyperspank}$-*tkmA′*-his_6-*′ptsG*(EIIBA^{Glc}), P_{penP}-*lacI*, *kanR*] Δ*ypqE* Δ*yyzE* Δ*levDEFG* Δ*mtlF::mrmrP* Δ*mdxRDEFGyvdJmalKLpgcM* Δ*ptsG* Δ*gamP::mrmrP* Δ*bglP::loxP* Δ*licA::loxP* Δ*gmuA::loxP* Δ*manP::loxP* Δ*fruA::loxP*	pKAM397 → KM455
KM873	*bglS*::[ter_{rrnB}-$P_{hyperspank}$-*tkmA′*-his_6, P_{penP}-*lacI*, *kanR*] Δ*ypqE* Δ*yyzE* Δ*levDEFG* Δ*mtlF*::*mrmrP* Δ*mdxRDEFGyvdJmalKLpgcM* Δ*ptsG* Δ*gamP*::*mrmrP* Δ*bglP*::*loxP* Δ*licA*::*loxP* Δ*gmuA*::*loxP* Δ*manP*::*loxP* Δ*fruA*::*loxP*	pKAM396 → KM455
KM877	*amyE*::[*ter*-P_{murR}-*lacZ*, *spcR*]	pKAM403 → KM0
KM878	*trpC2* Δ*murR*::*loxP* *amyE*::[*ter*-P_{murR}-*lacZ*, *spcR*]	pKAM403 → KM848
KM884	*bglS*::[ter_{rrnB}-$P_{hyperspank}$-*tkmA′*-his_6-*′gamP*(EIIBA^{Gam}), P_{penP}-*lacI*, *kanR*] Δ*ypqE* Δ*yyzE* Δ*levDEFG* Δ*mtlF*::*mrmrP* Δ*mdxRDEFGyvdJmalKLpgcM* Δ*ptsG* Δ*gamP*::*mrmrP* Δ*bglP*::*loxP* Δ*licA*::*loxP* Δ*gmuA*::*loxP* Δ*manP*::*loxP* Δ*fruA*::*loxP*	pKAM408 → KM455
KM885	*bglS*::[ter_{rrnB}-$P_{hyperspank}$-*tkmA′*-his_6-*′bglP*(EIIA^{Bgl}), P_{penP}-*lacI*, *kanR*] Δ*ypqE* Δ*yyzE* Δ*levDEFG* Δ*mtlF*::*mrmrP* Δ*mdxRDEFGyvdJmalKLpgcM* Δ*ptsG* Δ*gamP*::*mrmrP* Δ*bglP*::*loxP* Δ*licA*::*loxP* Δ*gmuA*::*loxP* Δ*manP*::*loxP* Δ*fruA*::*loxP*	pKAM409 → KM455
KM886	*bglS*::[ter_{rrnB}-$P_{hyperspank}$-*tkmA′*-his_6-*′manP*(EIIA^{Man}), P_{penP}-*lacI*, *kanR*] Δ*ypqE* Δ*yyzE* Δ*levDEFG* Δ*mtlF*::*mrmrP* Δ*mdxRDEFGyvdJmalKLpgcM* Δ*ptsG* Δ*gamP*::*mrmrP* Δ*bglP*::*loxP* Δ*licA*::*loxP* Δ*gmuA*::*loxP* Δ*manP*::*loxP* Δ*fruA*::*loxP*	pKAM410 → KM455
KM887	*bglS*::[ter_{rrnB}-$P_{hyperspank}$-*tkmA′*-his_6-*ypqE*, P_{penP}-*lacI*, *kanR*] Δ*ypqE* Δ*yyzE* Δ*levDEFG* Δ*mtlF*::*mrmrP* Δ*mdxRDEFGyvdJmalKLpgcM* Δ*ptsG* Δ*gamP*::*mrmrP* Δ*bglP*::*loxP* Δ*licA*::*loxP* Δ*gmuA*::*loxP* Δ*manP*::*loxP* Δ*fruA*::*loxP*	pKAM418 → KM455

KM896	Δ*sacX*	pKAM412 → KM0
KM907	Δ*sacX* Δ*sacP*::*loxP-ermC-loxP*	gDNA BKE38050 → KM896
KM908	Δ*sacX* Δ*sacP*::*loxP*	pJOE6732.1 → KM907
KM916	*bglS*::[ter_{rrnB}-$P_{hyperspank}$-*tkmA′*-his_6-*′ptsG*(EIIA^{Glc}), P_{penP}-*lacI*, *kanR*] Δ*ypqE* Δ*yyzE* Δ*levDEFG* Δ*mtlF*::*mrmrP* Δ*mdxRDEFGyvdJmalKLpgcM* Δ*ptsG* Δ*gamP*::*mrmrP* Δ*bglP*::*loxP* Δ*licA*::*loxP* Δ*gmuA*::*loxP* Δ*manP*::*loxP* Δ*fruA*::*loxP*	pKAM428 → KM455
KM917	*bglS*::[ter_{rrnB}-$P_{hyperspank}$-*tkmA′*-his_6-*′gamP*(EIIA^{Gam}), P_{penP}-*lacI*, *kanR*] Δ*ypqE* Δ*yyzE* Δ*levDEFG* Δ*mtlF*::*mrmrP* Δ*mdxRDEFGyvdJmalKLpgcM* Δ*ptsG* Δ*gamP*::*mrmrP* Δ*bglP*::*loxP* Δ*licA*::*loxP* Δ*gmuA*::*loxP* Δ*manP*::*loxP* Δ*fruA*::*loxP*	pKAM429 → KM455
KM918	Δ*glcK*::*loxP* *′ptsG*(EIICB, ΔEIIA) *bglS*::[ter_{rrnB}-$P_{hyperspank}$-*′ptsG*(EIIA^{Glc}), P_{penP}-*lacI*, *kanR*]	pKAM426 → KM445
KM919	Δ*glcK*::*loxP* *′ptsG*(EIICB, ΔEIIA) *bglS*::[ter_{rrnB}-$P_{hyperspank}$-*tkmA′*-his_6-*′ptsG*(EIIA^{Glc}), P_{penP}-*lacI*, *kanR*]	pKAM428 → KM445
KM920	Δ*glcK*::*loxP* *′ptsG*(EIIC, ΔEIIBA) *bglS*::[ter_{rrnB}-$P_{hyperspank}$-*tkmA′*-his_6-*′ptsG*(EIIBA^{Glc}), P_{penP}-*lacI*, *kanR*]	pKAM397 → KM446
KM921	Δ*glcK*::*loxP* *′ptsG*(EIIC, ΔEIIBA) *bglS*::[ter_{rrnB}-$P_{hyperspank}$-*′ptsG*(EIIBA^{Glc}), P_{penP}-*lacI*, *kanR*]	pKAM427 → KM446
KM930	Δ*ptsG* Δ*glcK*::*loxP* *bglS*::[ter_{rrnB}-$P_{hyperspank}$-*tkmA′*-his_6, P_{penP}-*lacI*, *kanR*]	pKAM396 → KM281
KM932	*amyE*::[*ter*-P_{sacX}-*lacZ*, *spcR*]	pKAM430 → KM0

KM933	*amyE*::[*ter*-P_{sacX}-*lacZ, spcR*] Δ*ypqE* Δ*yyzE* Δ*levDEFG* Δ*mtlF*::*mrmrP* Δ*mdxRDEFGyvdJmalKLpgcM* Δ*ptsG* Δ*gamP*::*mrmrP* Δ*bglP*::*loxP* Δ*licA*::*loxP* Δ*gmuA*::*loxP* Δ*manP*::*loxP*	pKAM430 → KM453
KM934	*amyE*::[*ter*-P_{sacX}-*lacZ, spcR*] *ptsG*$^+$ Δ*ypqE* Δ*yyzE* Δ*levDEFG* Δ*mtlF*::*mrmrP* Δ*mdxRDEFGyvdJmalKLpgcM* Δ*gamP*::*mrmrP* Δ*bglP*::*loxP* Δ*licA*::*loxP* Δ*gmuA*::*loxP* Δ*manP*::*loxP* Δ*fruA*::*loxP*	pKAM430 → KM790
KM935	*amyE*::[*ter*-P_{sacX}-*lacZ, spcR*] *levDEFG*$^+$ Δ*ypqE* Δ*yyzE* Δ*mtlF*::*mrmrP* Δ*mdxRDEFGyvdJmalKLpgcM* Δ*ptsG* Δ*gamP*::*mrmrP* Δ*bglP*::*loxP* Δ*licA*::*loxP* Δ*gmuA*::*loxP* Δ*manP*::*loxP* Δ*fruA*::*loxP*	pKAM430 → KM791
KM936	*amyE*::[*ter*-P_{sacX}-*lacZ, spcR*] *bglP*$^+$ Δ*ypqE* Δ*yyzE* Δ*levDEFG* Δ*mtlF*::*mrmrP* Δ*mdxRDEFGyvdJmalKLpgcM* Δ*ptsG* Δ*gamP*::*mrmrP* Δ*licA*::*loxP* Δ*gmuA*::*loxP* Δ*manP*::*loxP* Δ*fruA*::*loxP*	pKAM430 → KM792
KM937	*amyE*::[*ter*-P_{sacX}-*lacZ, spcR*] *licA*$^+$ Δ*ypqE* Δ*yyzE* Δ*levDEFG* Δ*mtlF*::*mrmrP* Δ*mdxRDEFGyvdJmalKLpgcM* Δ*ptsG* Δ*gamP*::*mrmrP* Δ*bglP*::*loxP* Δ*gmuA*::*loxP* Δ*manP*::*loxP* Δ*fruA*::*loxP*	pKAM430 → KM793
KM938	*amyE*::[*ter*-P_{sacX}-*lacZ, spcR*] *gamP*$^+$ Δ*ypqE* Δ*yyzE* Δ*levDEFG* Δ*mtlF*::*mrmrP* Δ*mdxRDEFGyvdJmalKLpgcM* Δ*ptsG* Δ*bglP*::*loxP* Δ*licA*::*loxP* Δ*gmuA*::*loxP* Δ*manP*::*loxP* Δ*fruA*::*loxP*	pKAM430 → KM794
KM939	*amyE*::[*ter*-P_{sacX}-*lacZ, spcR*] *mtlF*$^+$ Δ*ypqE* Δ*yyzE* Δ*levDEFG* Δ*mdxRDEFGyvdJmalKLpgcM* Δ*ptsG* Δ*gamP*::*mrmrP* Δ*bglP*::*loxP* Δ*licA*::*loxP* Δ*gmuA*::*loxP* Δ*manP*::*loxP* Δ*fruA*::*loxP*	pKAM430 → KM795

KM940	*amyE*::[*ter*-P$_{sacX}$-*lacZ, spcR*] *gmuA*$^{+}$ Δ*ypqE* Δ*yyzE* Δ*levDEFG* Δ*mtlF*::*mrmrP* Δ*mdxRDEFGyvdJmalKLpgcM* Δ*ptsG* Δ*gamP*::*mrmrP* Δ*bglP*::*loxP* Δ*licA*::*loxP* Δ*manP*::*loxP* Δ*fruA*::*loxP*	pKAM430 → KM796
KM941	*amyE*::[*ter*-P$_{sacX}$-*lacZ, spcR*] *manP*$^{+}$ Δ*ypqE* Δ*yyzE* Δ*levDEFG* Δ*mtlF*::*mrmrP* Δ*mdxRDEFGyvdJmalKLpgcM* Δ*ptsG* Δ*gamP*::*mrmrP* Δ*bglP*::*loxP* Δ*licA*::*loxP* Δ*gmuA*::*loxP* Δ*fruA*::*loxP*	pKAM430 → KM797
KM942	*amyE*::[*ter*-P$_{sacX}$-*lacZ, spcR*] *yvcA'*-P$_{ypqE}$-*ypqE-hisI* Δ*ypqE* Δ*yyzE* Δ*levDEFG* Δ*mtlF*::*mrmrP* Δ*mdxRDEFGyvdJmalKLpgcM* Δ*ptsG* Δ*gamP*::*mrmrP* Δ*bglP*::*loxP* Δ*licA*::*loxP* Δ*gmuA*::*loxP* Δ*manP*::*loxP* Δ*fruA*::*loxP*	pKAM430 → KM801
KM943	*amyE*::[*ter*-P$_{sacX}$-*lacZ, spcR*] *yvcA'*-P$_{yyzE}$-*yyzE-hisI* Δ*ypqE* Δ*yyzE* Δ*levDEFG* Δ*mtlF*::*mrmrP* Δ*mdxRDEFGyvdJmalKLpgcM* Δ*ptsG* Δ*gamP*::*mrmrP* Δ*bglP*::*loxP* Δ*licA*::*loxP* Δ*gmuA*::*loxP* Δ*manP*::*loxP* Δ*fruA*::*loxP*	pKAM430 → KM802
KM944	*amyE*::[*ter*-P$_{sacX}$-*lacZ, spcR*] Δ*ypqE* Δ*yyzE* Δ*levDEFG* Δ*mtlF*::*mrmrP* Δ*mdxRDEFGyvdJmalKLpgcM* Δ*ptsG* Δ*gamP*::*mrmrP* Δ*bglP*::*loxP* Δ*licA*::*loxP* Δ*gmuA*::*loxP* Δ*manP*::*loxP* Δ*fruA*::*loxP*	pKAM430 → KM455

[1] *Bacillus* Genetic Stock center

[2] genomic DNA

Table S2 Plasmids used in this study. Unless otherwise specified, PCRs were carried out using the genomic DNA of KM0.

Plasmid	Genotype	Vector (cut)	Insert (cut)	Reference or description
pBR322	*ori*$_{pBR322}$, *bla*, *tetR*, *lacZ*α			(5)
pDG1730	*ermC, bla, amyE'-spcR-amyE'*			(6)
pDR111	*ori*$_{colE1}$, *amyE*-[*spcR*, *ter*$_{rrnB}$-P$_{hyperspank}$-P$_{penP}$-*lacI*]-*amyE*, *bla*			D. Rudner (unpublished)
pHM30	*ori*$_{pUC18}$, *bla, 'hisF-hisI'-spcR-'yvcA-yvcB'*			(7)
pHM31	*ori*$_{pUC18}$, *bla, 'hisF-hisI-ter-'yvcA-yvcB'*			(7)
pJOE4786.1	*ori*$_{pUC18}$, *bla*, *ter-'lacI-lacZα-ter*			(8)
pJOE6089.4	*ori*$_{pUC18}$, *rop*, *bla*, *rha*P$_{BAD}$-*eGFP-strep* tag-*rrnB*			(9)
pJOE6577.1	*ori*$_{pUC18}$, *bla*, *spcR*, *'manR-manP-ermC-yjdF*			(10)
pJOE6732.1	*ori*$_{pAMβ1}$, *repDE*, P$_{xyl}$-*cre*$_{P1}$, *spc*, ori$_{pUC18}$, *bla*			J. Altenbuchner (unpublished)
pJOE6743.1	*ori*$_{pUC18}$, *bla*, P$_{manP}$-*manP*, *spcR*			(10)
pJOE7644.2	*ori*$_{pUC18}$, *bla*, P$_{manP}$-*manP*, *spcR*, *'manR-ctaO'*			(10)
pJOE8525.2	*ori*$_{pUC18}$, *bla*, P$_{manP}$-*manP*, *spcR*, *'clpP-yvdD'*	pJOE6743.1 (BamHI)	PCR s9475-s9476 from pKAM238 (BamHI)	*'clpP-yvdD'* → pJOE6743.1
pJOE8999.1	*ori*$_{pUC18}$, *ori*$_{pE194ts}$, *kanR*, P$_{manP}$-*cas9*, P$_{vanP}$-*lacPOZ'-gRNA-ter*$_{oop}$			(11)
pMUTIN-FLAG	*ori*$_{colE1}$, *bla*, *ermC*, *ter*-P$_{spac}$-FLAG-*ter*-P$_{penP}$-*lacI*			BGSC
pMW312.2	*ori*$_{pUC18}$, *'glcT'-ermC-ptsHI'*, *bla*, *spcR*			(12)
pMW373.3	*ori*$_{pUC18}$, *'glcT*-P$_{ptsGHI}$- *ptsHI'*, *bla*, *spcR*			(12)
pMW521.1	*ori*$_{pE194ts}$, *ori*$_{pUC18}$, *spcR*			(13)
pMW850.2	*ori*$_{pUC18}$, *rop*, *cer*, *rha*P$_{BAD}$-*ptsH-his*$_6$-*rrnB*, *bla*			(14)
pMW851.2	*ori*$_{pUC18}$, *rop*, *cer*, *rha*P$_{BAD}$-*ptsI-his*$_6$-*rrnB*, *bla*			(14)

Plasmid	Genotype	Vector (cut)	Insert (cut)	Reference or description
pUC18	*ori*$_{\mathrm{pUC18}}$, *bla*, *lacZ*α			(15)
pYFPbglS	*ori*$_{\mathrm{colE1}}$, *bla*, f1(+) origin, *bglS'*-[LIC-*yfp*, *kanR*]-*'bglS*			(16)
pKAM020	*ori*$_{\mathrm{pUC18}}$, *mtlA'-mroxP-cat-mroxP*, *bla*	pKAM19 (BamHI-XhoI)	PCR s6759-s6760 (BamHI-XhoI)	*mtlA'* → pKAM19
pKAM041	*ori*$_{\mathrm{pUC18}}$, *bla*, *ter-'trpD-trpC-trpF'-ter*			(4)
pKAM051	*ori*$_{\mathrm{pUC18}}$, *bla*, *ter-'yfkQ*-P$_{treP}$-*ter*	pJOE4786.1 (SmaI)	PCR s9166-s9167	*'yfkQ*-P$_{treP}$ → pJOE4786.1
pKAM052	*ori*$_{\mathrm{pUC18}}$, *bla*, *ter-'treA-ter*	pJOE4786.1 (SmaI)	PCR s9168-s9169	*'treA* → pJOE4786.1
pKAM063	*ori*$_{\mathrm{pUC18}}$, *bla*, *ter-'gamA-ter*	pJOE4786.1 (SmaI)	PCR s9386-s9387	*'gamA* → pJOE4786.1
pKAM064	*ori*$_{\mathrm{pUC18}}$, *bla*, *ter-gltP'-ter*	pJOE4786.1 (SmaI)	PCR s9388-s9389	*gltP'* → pJOE4786.1
pKAM065	*ori*$_{\mathrm{pUC18}}$, *'gamA-mroxP-cat-mroxP*, *bla*	pKAM19 (BamHI-XhoI)	pKAM063 (BamHI-XhoI)	*'gamA* → pKAM19
pKAM073	*ori*$_{\mathrm{pUC18}}$, *bla*, *ter*-P$_{ypqE}$-*ter*	pJOE4786.1 (SmaI)	PCR s9485-s9486	P$_{ypqE}$ → pJOE4786.1
pKAM095	*ori*$_{\mathrm{pUC18}}$, *bla*, *ter*-P$_{glcR}$-*ter*	pJOE4786.1 (SmaI)	PCR s9990-s9991	P$_{glcR}$ → pJOE4786.1
pKAM0131	*ori*$_{\mathrm{pUC18}}$, *bla*, *ter*-P$_{ypqE}$-*ypqE-ter*	pJOE4786.1 (SmaI)	PCR s11135-s11136	P$_{ypqE}$-*ypqE* → pJOE4786.1
pKAM0132	*ori*$_{\mathrm{pUC18}}$, *bla*, *ter*-P$_{yyzE}$-*yyzE-ter*	pJOE4786.1 (SmaI)	PCR s11137-s11138	P$_{yyzE}$-*yyzE* → pJOE4786.1
pKAM0146	*ori*$_{\mathrm{pUC18}}$, *bla*, *ter-'ptsG* (EIIBA)-*ter*	pJOE4786.1 (SmaI)	PCR s11467-s11468	*'ptsG* (EIIBA) → pJOE4786.1

Plasmid	Genotype	Vector (cut)	Insert (cut)	Reference or description
pKAM0147	*ori*$_{pUC18}$, *bla*, *ter*-′*gamP* (EIIBA)-*ter*	pJOE4786.1 (SmaI)	PCR s11569-s11570	′*gamP* (EIIBA) → pJOE4786.1
pKAM0148	*ori*$_{pUC18}$, *bla*, *ter*-′*bglP* (EIIA)-*ter*	pJOE4786.1 (SmaI)	PCR s11571-s11572	′*bglP* (EIIA) → pJOE4786.1
pKAM0149	*ori*$_{pUC18}$, *bla*, *ter*-′*manP* (EIIA)-*ter*	pJOE4786.1 (SmaI)	PCR s11573-s11574	′*manP* (EIIA) → pJOE4786.1
pKAM0150	*ori*$_{pUC18}$, *bla*, *ter*-*ypqE*-*ter*	pJOE4786.1 (SmaI)	PCR s9718-s11543	*ypqE* → pJOE4786.1
pKAM0151	*ori*$_{pUC18}$, *bla*, *ter*-P$_{murQ}$-*yyzE*-*ter*	pJOE4786.1 (SmaI)	PCR s11533-s11534	P$_{murQ}$ → pJOE4786.1
pKAM0156	*ori*$_{pUC18}$, *bla*, *ter*-′*epr*-*sacY*′-*ter*	pJOE4786.1 (SmaI)	Primary PCRs s11618-s11619 and s11620-s11621 Fusion PCR s11618-s11621	*epr*-*sacY*′ → pJOE4786.1
pKAM0157	*ori*$_{pUC18}$, *bla*, *ter*-*ypqE*-*ter*	pJOE4786.1 (SmaI)	PCR s11697-s11698	*ypqE* → pJOE4786.1
pKAM0161	*ori*$_{pUC18}$, *bla*, *ter*-′*ptsG* (EIIA)-*ter*	pJOE4786.1 (SmaI)	PCR s11808-s11244	′*ptsG* (EIIA) → pJOE4786.1
pKAM0162	*ori*$_{pUC18}$, *bla*, *ter*-′*ptsG* (EIIBA)-*ter*	pJOE4786.1 (SmaI)	PCR s11809-s11244	′*ptsG* (EIIBA) → pJOE4786.1
pKAM0163	*ori*$_{pUC18}$, *bla*, *ter*-′*ptsG* (EIIA)-*ter*	pJOE4786.1 (SmaI)	PCR s11810-s11244	′*ptsG* (EIIA) → pJOE4786.1
pKAM0164	*ori*$_{pUC18}$, *bla*, *ter*-′*gamP* (EIIA)-*ter*	pJOE4786.1	PCR	′*gamP* (EIIA) → pJOE4786.1

Plasmid	Genotype	Vector (cut)	Insert (cut)	Reference or description
		(SmaI)	s11811-s11570	
pKAM0165	ori_{pUC18}, *bla*, *ter*-P_{sacX}-*ter*	pJOE4786.1 (SmaI)	PCR s11856-s11857	$P_{sacX} \rightarrow$ pJOE4786.1
pKAM19	ori_{pUC18}, *mroxP-cat-mroxP*, *bla*			(17)
pKAM20	ori_{pUC18}, *mroxP-ermC-mroxP*, *bla*	pKAM19 (SpeI)	PCR s6512-s6513 from pMW312.2 (SpeI)	*ermC* $\rightarrow$ pKAM19
pKAM47	ori_{pUC18}, *mtlA′-mroxP-cat-mroxP-mtlD′*, *bla*	pKAM020 (NheI-SacI)	PCR s6761-s6762 (NheI-SacI)	*mtlD′* $\rightarrow$ pKAM020
pKAM114	ori_{pBR322}, ori^{+}_{pUB110}, rep_{pUB110}, *spcR*, *ter*-P_{mtlA}-TIR_{gsiB}-*eGFP-ter*			(4)
pKAM123	ori_{pBR322}, *bla*, *amyE′*-[*ter*-P_{mtlA}-*lacZ-spcR*]-*amyE′*, *ermC*			(17)
pKAM152	ori_{pUC18}, *bla*, *′ptsG* (EIICBA)-P_{ptsHI}	pUC18 (XmaI)	PCR s7582-s7583 (XmaI)	*′ptsG* (EIICBA)-$P_{ptsHI} \rightarrow$ pUC18
pKAM156	ori_{pUC18}, *bla*, *′ptsG* (EIICB-linkerEIIA)-P_{ptsHI}	pUC18 (XmaI)	Primary PCR s7582-s7589 and s7590-s7583 Fusion PCR s7582-s7583 (XmaI)	*′ptsG* (EIICB-linkerEIIA)-$P_{ptsHI} \rightarrow$ pUC18
pKAM157	ori_{pUC18}, *bla*, *′ptsG* (EIICBlinker-EIIA)-P_{ptsHI}	pUC18 (XmaI)	Primary PCR s7582-s7591 and s7592-s7583 Fusion PCR s7582-s7583 (XmaI)	*′ptsG* (EIICBlinker-EIIA)-$P_{ptsHI} \rightarrow$ pUC18

**Plasmid**	**Genotype**	**Vector (cut)**	**Insert (cut)**	**Reference or description**
pKAM158	ori_{pUC18}, *bla*, *'ptsG* (EIICB-EIIA)-P_{ptsHI}	pUC18 (XmaI)	Primary PCR s7582-s7593 and s7592-s7583 Fusion PCR s7582-s7583 (XmaI)	*'ptsG* (EIICB-EIIA)-P_{ptsHI} → pUC18
pKAM171	ori_{pUC18}, *bla*, *'ptsG* (EIICBA)-P_{ptsHI}-*ptsHI'*	pKAM152 (AgeI-BamHI)	PCR s8182-s8183 (AgeI-BamHI)	*'ptsG* (EIIA)-P_{ptsHI}-*ptsHI'* → pKAM152
pKAM172	ori_{pUC18}, *bla*, *'ptsG'* (EIIC)-*ermC*-*ptsHI'*	pKAM171 (BglII-NheI)	PCR s7712-s7713 (NheI-BglII) using pKAM20 as template	*ermC* → pKAM171
pKAM173	ori_{pUC18}, *bla*, *' ptsG* (EIICB-linkerEIIA)-P_{ptsHI}-*ptsHI'*	pKAM156 (AgeI-BamHI)	PCR s8182-s8183 (AgeI-BamHI)	*'ptsG* (EIIA)-P_{ptsHI}-*ptsHI'* → pKAM156
pKAM174	ori_{pUC18}, *bla*, *'ptsG* (EIICBlinker-EIIA)-P_{ptsHI}-*ptsHI'*	pKAM157 (AgeI-BamHI)	PCR s8182-s8183 (AgeI-BamHI)	*'ptsG* (EIIA)-P_{ptsHI}-*ptsHI'* → pKAM157
pKAM175	ori_{pUC18}, *bla*, *'ptsG* (EIICB-EIIA)-P_{ptsHI}-*ptsHI'*	pKAM158 (AgeI-BamHI)	PCR s8182-s8183 (AgeI-BamHI)	*'ptsG* (EIIA)-P_{ptsHI}-*ptsHI'* → pKAM158
pKAM205	ori_{colE1}, *bla*, *ermC*, *ter*-P_{spac}-*mtlR*-FLAG-*ter*-P_{penP}-*lacI*	pMUTIN-FLAG (PsiI-KpnI)	PCR s8327-s8328 (PsiI-KpnI)	*mtlR* → pMUTIN-FLAG
pKAM206	ori_{colE1}, f1(+) origin, *bla*, *bglS'*-[ter_{rrnB}-P_{spac}-*mtlR*-*ter*-P_{penP}-*lacI*-*kanR*]-*'bglS*	pKAM216 (BglII-PstI)	pKAM205 (PstI-BamHI)	*ter*-P_{spac}-*mtlR*-*ter*-P_{penP}-*lacI*-*ter* → pKAM205

Plasmid	Genotype	Vector (cut)	Insert (cut)	Reference or description
pKAM216	*ori*$_{colE1}$, f1(+) origin, *bla*, *bglS'*-[Multiple cloning site-*kanR*]-'*bglS*	pYFPbglS (PstI-EcoRI)	Hybridization of s8431-s8432 (PstI-EcoRI)	Multiple cloning site → pYFPbglS
pKAM224	*ori*$_{pE194ts}$, *ori*$_{pUC18}$, *spcR*, *'yppG-ypqA-yprA'*	pMW521.1 (BamHI-XmaI)	Primary PCRs s8629-s8630 and s8631-s8632 Fusion PCR s8629-s8632 (BamHI-XmaI)	*'yppG-ypqA-yprA'* → pMW521.1
pKAM234	*ori*$_{pE194ts}$, *ori*$_{pUC18}$, *spcR*, '*levR-sacC*'	pMW521.1 (BamHI-XmaI)	Primary PCRs s8733-s8734 and s8735-s8736 Fusion PCR s8733-s8736 (BamHI-XmaI)	'*levR-sacC*' → pMW521.1
pKAM235	*ori*$_{pE194ts}$, *ori*$_{pUC18}$, *spcR*, '*bglA-yydK*'	pMW521.1 (BamHI-PmeI)	PCR s8737-s8817 (BamHI-BsaI) PCR s8818-s8740 (BsaI-SmaI)	'*bglA* + *yydK*' → pMW521.1
pKAM238	*ori*$_{pE194ts}$, *ori*$_{pUC18}$, *spcR*, '*clpP-yvdD*'	pMW521.1 (BamHI-PmeI)	PCR s8761-s8762 (PmeI-BsaI) PCR s8763-s8764 (BsaI-BamHI)	'*clpP* + *yvdD*' → pMW521.1
pKAM239	*ori*$_{pE194ts}$, *ori*$_{pUC18}$, *spcR*, '*ilvK-yxzF-aag'*	pMW521.1 (BamHI-PmeI)	PCR s8765-s8766 (SmaI-BsaI)	'*ilvK* + *yxzF-aag*'- → pMW521.1

Plasmid	Genotype	Vector (cut)	Insert (cut)	Reference or description
			PCR s8767-s8768 (BsaI-BamHI)	
pKAM241	*ori*$_{\text{pBR322}}$, *bla*, *amyE′*-[*spcR*]-*amyE′*, *ermC*	pDG1730 (NdeI → Klenow fill in → religate)		Removing the NdeI restriction site
pKAM243	*ori*$_{\text{pBR322}}$, *bla*, *amyE′*-[*ter*-P$_{mtlA}$-*lacZ*-*spcR*]-*′amyE*, *ermC*	pKAM241 (HindIII-EcoRI)	pKAM123 (HindIII-EcoRI)	*ter*-P$_{mtlA}$-*lacZ* → pKAM241
pKAM246	*ori*$_{\text{colE1}}$, f1(+) origin, *bla*, *bglS′*-[*ter*$_{rrnB}$-P$_{hyperspank}$-*mtlR*-*ter*-P$_{penP}$-*lacI*-*kanR*]-*′bglS*	pKAM206 (PacI-AflII)	PCR s8900-s8901 from pDR111 (PacI-AflII)	P$_{hyperspank}$→pKAM206
pKAM262	*ori*$_{\text{pBR322}}$, *rop*, *tet*, *bla*	pBR322 (NdeI → Klenow fill in → religate)		Removing the NdeI restriction site
pKAM263	*ori*$_{\text{pBR322}}$, *rop*, *bla*, *amyE′*-[*ter*-P$_{mtlA}$-*lacZ*-*spcR*]-*′amyE*, *ermC*	pKAM262 (ScaI-MscI)	pKAM243 (ScaI-MscI)	*bla*-*amyE*'-*ter*-P$_{mtlA}$-*lacZ*-*spcR*-*amyE*'-*ermC* → pKAM262
pKAM268	*ori*$_{\text{pUC18}}$, *yfkQ*-P$_{treP}$ -*mroxP*-*cat*-*mroxP*, *bla*	pKAM19 (BamHI-XhoI)	pKAM051 (BamHI-XhoI)	'*yfkQ*-P$_{treP}$ → pKAM19
pKAM269	*ori*$_{\text{pUC18}}$, *yfkQ*-P$_{treP}$ -*mroxP*-*cat*-*mroxP*-*treA′*, *bla*	pKAM268 (EcoRI-SacI)	pKAM052 (EcoRI-SacI)	*treA*' → pKAM268
pKAM286	*ori*$_{\text{pUC18}}$, '*gamA*-*mroxP*-*cat*-*mroxP*-*gltP*', *bla*	pKAM065 (NheI-SacI)	pKAM064 (NheI-SacI)	*gltP*' → pKAM065
pKAM292	*ori*$_{\text{pBR322}}$, *rop*, *bla*, *ermC*, *amyE′*-[*ter*-P$_{ypqE}$-*lacZ*-*spcR*]-*′amyE*	pKAM312	pKAM073	P$_{ypqE}$ → pKAM312

Plasmid	Genotype	Vector (cut)	Insert (cut)	Reference or description
pKAM299	*ori*$_{pBR322}$, *ori*$^{+}_{pUB110}$, *rep*$_{pUB110}$, *spcR*, *ter*-P$_{ypqE}$-TIR$_{gsiB}$-*eGFP*-*ter*	(AgeI-NdeI) pKAM114 (AgeI-NdeI)	(AgeI-NdeI) pKAM073 (AgeI-NdeI)	P$_{ypqE}$ → pKAM114
pKAM306	*ori*$_{pUC18}$, *bla*, *ptsG′* (EIICB)-P$_{ptsHI}$-*ptsHI*	pKAM175 site directed mutagenesis s9824-s9825		*ptsG′* (EIICB)-P$_{ptsHI}$-*ptsHI*
pKAM307	*ori*$_{pUC18}$, *bla*, *ptsG′* (EIIC)-P$_{ptsHI}$-*ptsHI*	pKAM175 site directed mutagenesis s9824-s9826		*ptsG′* (EIIC)-P$_{ptsHI}$-*ptsHI*
pKAM308	*ori*$_{pUC18}$, *bla*, *ptsG′* (EIIC-EIIA)-P$_{ptsHI}$-*ptsHI*	pKAM175 site directed mutagenesis s9826-s9827		*ptsG′* (EIIC-EIIA)-P$_{ptsHI}$-*ptsHI*
pKAM312	*ori*$_{pBR322}$, *rop*, *ermC*, *bla*, *amyE′*-[*ter*-P$_{glcR}$-*lacZ*-*spcR*]-*′amyE*	pKAM263 (NheI-NdeI)	pKAM095 (NheI-NdeI)	P$_{glcR}$ → pKAM263
pKAM374	*ori*$_{pUC18}$, *bla*, *'hisF*-*hisI*-*ter*-[P$_{ypqE}$-*ypqE*]-*′yvcA*-*yvcB′*	pHM31 (NheI-XmaI)	pKAM0131 (NheI-XmaI)	P$_{ypqE}$-*ypqE* → pHM31
pKAM375	*ori*$_{pUC18}$, *bla*, *'hisF*-*hisI*-*ter*-[P$_{yyzE}$-*yyzE*]-*′yvcA*-*yvcB′*	pHM31 (NheI-XmaI)	pKAM0132 (NheI-XmaI)	P$_{yyzE}$-*yyzE* → pHM31
pKAM379	*ori*$_{pUC18}$, *bla*, *'hisF*-*hisI*-*ter*-[*′levR*-P$_{lev}$-*levD*]-*′yvcA*-*yvcB′*	pHM31 (NheI-BamHI)	PCR s11245-s11246 (NheI-BamHI)	*′levR*-P$_{lev}$-*levD* → pHM31

Plasmid	Genotype	Vector (cut)	Insert (cut)	Reference or description
pKAM396	*ori*$_{\text{colE1}}$, f1(+) origin, *bla*, *bglS′*-[*ter*$_{rrnB}$-P$_{hyperspank}$-*tkmA′*-*his*$_{6}$-*ter*-P$_{penP}$-*lacI*-*kanR*]-*′bglS*	pKAM246 (BglII-XmaI)	PCR s11465-s11466 (BglII-XmaI)	*tkmA′*-*his*$_{6}$ → pKAM246
pKAM397	*ori*$_{\text{colE1}}$, f1(+) origin, *bla*, *bglS′*-[*ter*$_{rrnB}$-P$_{hyperspank}$-*tkmA′*-*his*$_{6}$-*′ptsG* (EIIBA)-*ter*-P$_{penP}$-*lacI*-*kanR*]-*′bglS*	pKAM396 (BsrGI-XmaI)	pKAM0146 (BsrGI-XmaI)	*′ptsG* (EIIBA) → pKAM396
pKAM401	*ori*$_{\text{pUC18}}$, *rop*, *bla*, *rha*P$_{\text{BAD}}$-*ypqE*-*strep* tag-*rrnB*	pJOE6089.4 (NdeI-BamHI)	pKAM0150 (NdeI-BamHI)	*ypqE* → pJOE6089.4
pKAM403	*ori*$_{\text{pBR322}}$, *rop*, *ermC*, *bla*, *amyE′*-[*ter*-P$_{murQ}$-*lacZ*-*spcR*]-*′amyE*	pKAM312 (AgeI-NdeI)	pKAM0151 (AgeI-NdeI)	P$_{murQ}$ → pKAM312
pKAM408	*ori*$_{\text{colE1}}$, f1(+) origin, *bla*, *bglS′*-[*ter*$_{rrnB}$-P$_{hyperspank}$-*tkmA′*-*his*$_{6}$-*′gamP* (EIIBA)-*ter*-P$_{penP}$-*lacI*-*kanR*]-*′bglS*	pKAM396 (BsrGI-XmaI)	pKAM0147 (BsrGI-XmaI)	*gamP* (EIIBA) → pKAM396
pKAM409	*ori*$_{\text{colE1}}$, f1(+) origin, *bla*, *bglS′*-[*ter*$_{rrnB}$-P$_{hyperspank}$-*tkmA′*-*his*$_{6}$-*′bglP* (EIIA)-*ter*-P$_{penP}$-*lacI*-*kanR*]-*′bglS*	pKAM396 (BsrGI-XmaI)	pKAM0148 (BsrGI-XmaI)	*bglP* (EIIA) → pKAM396
pKAM410	*ori*$_{\text{colE1}}$, f1(+) origin, *bla*, *bglS′*-[*ter*$_{rrnB}$-P$_{hyperspank}$-*tkmA′*-*his*$_{6}$-*′manP* (EIIA)-*ter*-P$_{penP}$-*lacI*-*kanR*]-*′bglS*	pKAM396 (BsrGI-XmaI)	pKAM0149 (BsrGI-XmaI)	*manP* (EIIA) → pKAM396
pKAM411	*ori*$_{\text{pUC18}}$, *ori*$_{\text{pE194ts}}$, *kanR*, P$_{manP}$-*cas9*, P$_{vanP}$-*seed*$_{sacX}$-*gRNA*-*ter*$_{oop}$	pJOE8999.1 (BsaI)	Hybridization of s11616-s11617	*seed*$_{sacX}$ → pJOE8999.1
pKAM412	*ori*$_{\text{pUC18}}$, *ori*$_{\text{pE194ts}}$, *kanR*, P$_{manP}$-*cas9*, P$_{vanP}$-*seed*$_{sacX}$-*gRNA*-*ter*$_{oop}$, *'epr-sacY'*	pKAM411 (SmaI → SfiI)	pKAM0156 (SfiI)	*'epr-sacY'* → pKAM411
pKAM418	*ori*$_{\text{colE1}}$, f1(+) origin, *bla*, *bglS′*-[*ter*$_{rrnB}$-P$_{hyperspank}$-*tkmA′*-*his*$_{6}$-*ypqE*-*ter*-P$_{penP}$-*lacI*-*kanR*]-*′bglS*	pKAM396 (BsrGI-XmaI)	pKAM0157 (BsrGI-XmaI)	*ypqE* → pKAM396
pKAM426	*ori*$_{\text{colE1}}$, f1(+) origin, *bla*, *bglS′*-[*ter*$_{rrnB}$-P$_{hyperspank}$-*′ptsG* (EIIA)-*ter*-P$_{penP}$-*lacI*-*kanR*]-*′bglS*	pKAM396 (BglII-XmaI)	pKAM0161 (BamHI-XmaI)	*′ptsG* (EIIA) → pKAM396

Plasmid	Genotype	Vector (cut)	Insert (cut)	Reference or description
pKAM427	*ori*$_{\text{colE1}}$, f1(+) origin, *bla*, *bglS′*-[*ter*$_{rrnB}$-P$_{hyperspank}$-*′ptsG* (EIIBA)-*ter*-P$_{penP}$-*lacI*-*kanR*]-*′bglS*	pKAM396 (BglII-XmaI)	pKAM0162 (BamHI-XmaI)	*′ptsG* (EIIBA) $\rightarrow$ pKAM396
pKAM428	*ori*$_{\text{colE1}}$, f1(+) origin, *bla*, *bglS′*-[*ter*$_{rrnB}$-P$_{hyperspank}$-*tkmA′*-*his*$_6$-*′ptsG* (EIIA)-*ter*-P$_{penP}$-*lacI*-*kanR*]-*′bglS*	pKAM396 (BsrGI-XmaI)	pKAM0163 (BsrGI-XmaI)	*′ptsG* (EIIA) $\rightarrow$ pKAM396
pKAM429	*ori*$_{\text{colE1}}$, f1(+) origin, *bla*, *bglS′*-[*ter*$_{rrnB}$-P$_{hyperspank}$-*tkmA′*-*his*$_6$-*′gamP* (EIIA)-*ter*-P$_{penP}$-*lacI*-*kanR*]-*′bglS*	pKAM396 (BsrGI-XmaI)	pKAM0164 (BsrGI-XmaI)	*′gamP* (EIIA) $\rightarrow$ pKAM396
pKAM430	*ori*$_{\text{pBR322}}$, *rop*, *ermC*, *bla*, *amyE′*-[*ter*-P$_{sacX}$-*lacZ*-*spcR*]-*′amyE*	pKAM312 (AgeI-NdeI)	pKAM0165 (AgeI-BfaI)	P$_{sacX}$ $\rightarrow$ pKAM312

TABLE S3 Oligonucleotides used in this study.

Name	Sequence (5′→ 3′)	Application
**s5621**	AAA AAA GGC GCC TGG ATT ACC GTC TCA TCG	Amplification of *glcT-ptsGHI*
**s5624**	GTC GCA ATC ATA GGG AAC AT	Amplification of *glcT-ptsGHI*
**s6345**	AAA AAA CAT ATG TTA AAA TGA TGG CGT GCA ACG	Complementation of *mtlF*
**s6512**	AAA AAA ACTAGT CCC GCG GAT GCA TAT GAT C	Amplification of *ermC*
**s6513**	AAA AAA ACTAGT CAA TTG AAT CGA TTC ACA AAA	Amplification of *ermC*
**s6759**	AAA AAA GGATCC CAT CCA TTT CTT CGG AGG	Complementation/ Deletion of *mtlF*
**s6760**	AAA AAA CTCGAG GAC AAT CAC TCT CTT TCT ATA AGA T	Deletion of *mtlF*
**s6761**	AAA AAA GCTAGC CAT TTT CAA CGA GGT GAA CT	Deletion of *mtlF*
**s6762**	AAA AAA GAGCTC TTT GAC CGT TTT GAG TCC G	Deletion of *mtlF*
**s7582**	AAA AAA CCC GGG GAC TTT CAT TCA TGG TCA TGC	Amplification of *ptsG*
**s7583**	AAA AAA CCCGGG CCA TTT TTA TCA TTC TCC TTT TA	Amplification of *ptsG*
**s7589**	AGG CAT AAG AAT TGA CCT CCT TTA TCT AGG CTT GCG TCC CG	Mutation of *ptsG*
**s7590**	TAA AGG AGG TCA ATT CTT ATG CCT GAG CCG AAA ACA TCT G	Mutation of *ptsG*
**s7591**	GTC CAT AAG AAT TGA CCT CCT TTA AGG AAC ATC CGT AAT TGG GTG	Mutation of *ptsG*
**s7592**	TAA AGG AGG TCA ATT CTT ATG GAC CAA GTC TTC TCA GGG AAA A	Mutation of *ptsG*
**s7593**	GTC CAT AAG AAT TGA CCT CCT TTA TCT AGG CTT GCG TCC CG	Mutation of *ptsG*
**s7711**	AAA AAA CCC GGG GTC AAT AAT GAC ATC GCC AT	Amplification of *ptsG*
**s7712**	AAA AAA GCT AGC CCC GCG GAT GCA TAT GAT C	Amplification of *ermC*
**s7713**	AAA AAA AGA TCT CAA TTG AAT CGA TTC ACA AAA	Amplification of *ermC*
**s8044**	GGA AGA GGC AGC ATA TTT G	Complementation of *manP*
**s8182**	AAG GAA TTG TCG TAT CAC CGG T	Amplification of *ptsGHI*
**s8183**	AAA AAA GGA TCC GTC AAT AAT GAC ATC GCC AT	Amplification of *ptsGHI*
**s8327**	CCC CCC GGT ACC AGATCT AGG AGG TCT TTT TTA TGT ATA TGA	amplification of *mtlR*
**s8328**	TTT TTT TTA TAA CCCGGG TTA CAG TAT GTT TTT TTC TTT CAT C	amplification of *mtlR*
**s8431**	AAA AAA CTG CAG GCT AGC GCT ATC CCG GGG AGA TCT GAA TTC AAA AAA	Construction of a multiple cloning site

**s8432**	TTT TTT GAA TTC AGA TCT CCC CGG GAT AGC GCT AGC CTG CAG TTT TTT	Construction of a multiple cloning site
**s8484**	Cy5-CTT GCC GTA GGT GGC ATC	Primer extension
**s8629**	AAA AAA GGA TCC CTT CTG GCA AAA ACA ATG GT	Deletion of *ypqE*
**s8630**	ATG AGA AGG GAG AAA ATA CAT TGT AAG CAG GGC GTA TGC CTT GC	Deletion of *ypqE*
**s8631**	GCA AGG CAT ACG CCC TGC TTA CAA TGT ATT TTC TCC CTT CTC AT	Deletion of *ypqE*
**s8632**	AAA AAA CCC GGG CCT CGG CAA ATG GGC TAT	Deletion of *ypqE*
**s8659**	AAA AAA CCC GGG TCT GTG CTT CTT TTT CGC T	Complementation of *manP*
**s8733**	TTT TTT GGA TCC TTC ACC TTC GGA CAT TCT T	Deletion of *levDEFG*
**s8734**	CTC TTT CCT TCA CAC ACC TTA CAT ATC TAT TGC TCC TTT CCT	Deletion of *levDEFG*
**s8735**	AGG AAA GGA GCA ATA GAT ATG TAA GGT GTG TGA AGG AAA GAG	Deletion of *levDEFG*
**s8736**	GGG GGG CCC GGG TTT GGA ATG ACA GGG TTG	Deletion of *levDEFG*
**s8737**	AAA AAA GGA TCC GAA ATA GCG CTG CCT CAT	Deletion of *yyzE*
**s8740**	AAA AAA CCC GGG CGA GTA ATC AAA CGG CTG	Deletion of *yyzE*
**s8761**	AAA AAA GTT TAA ACA GTC ATT GAA CAA ACG AAC C	Deletion of *mdx* operon
**s8762**	AAA AAA GGT CTC ATG TGA AAT GAA AAA AAC CTG CA	Deletion of *mdx* operon
**s8763**	ATA TAT GGT CTC TCA CAT TTT CAT CCC CTC CTT A	Deletion of *mdx* operon
**s8764**	AAA AAA GGA TCC AAC CAT TTG TGT ATT TGC GG	Deletion of *mdx* operon
**s8765**	AAA AAA CCCGGG GGC TGC AAT GGT CTA TCA	Deletion of *licBCAH*
**s8766**	AAA AAA GGT CTC ATG TAA AAA AAG CCG GCC TGT AA	Deletion of *licBCAH*
**s8767**	ATA TAT GGT CTC TTA CAT GAG AAA AAC CTC CTA ACT A	Deletion of *licBCAH*
**s8768**	AAA AAA GGA TCC CTG GGC TGC CTT CTT GTA	Deletion of *licBCAH*
**s8817**	ATA TAT GGT CTC ATG TGA GGA GGA ATT AAA CAT GG	Deletion of *yyzE*
**s8818**	ATA TAT GGT CTC TCA CAT GAC AAC CGT AAT GAC G	Deletion of *yyzE*
**s8900**	CGA TGA TTA ATT AAT TCA GAA C	Amplification of $P_{hyperspank}$
**s8901**	AAA AAA CTT AAG AAT TGT TAT CCG CTC ACA A	Amplification of $P_{hyperspank}$
**s9166**	AAA AAA GGA TCC GTA CGT GTC GTC TTC GAT AA	Deletion of *treP*
**s9167**	ATC GTA CTC GAG CCG TAT CCG TTT TTA TAA CTT	Deletion of *treP*

**s9168**	AAA AAA GAA TTC CAA GTG GGG AGC GGG AC	Deletion of *treP*
**s9169**	AAAAAA GAG CTC CGC CGT TAG GAT AGT CAA C	Deletion of *treP*
**s9386**	AAA AAA GGA TCC ATC GCC GAG CAC TAC GA	Deletion of *gamP*
**s9387**	AAA AAA CTC GAG CAT GAC AGT CTC CTT TTA TTG	Deletion of *gamP*
**s9388**	AAA AAA GCT AGC TAA AAA AGT CCC CCC TGC	Deletion of *gamP*
**s9389**	AAA AAA GAG CTC AGC TGT AGA TTC AAA AAA CTT C	Deletion of *gamP*
**s9475**	TTT TTT GGA TCC AAC CAT TTG T	Deletion of *mdx* operon
**s9476**	TTT TTT GGA TCC GCG GGG AAA GAG CGT	Deletion of *mdx* operon
**s9485**	AAA AAA ACC GGT TGC CTC CGT TAT GAT TTC TT	Amplification of P_{ypqE}
**s9486**	AAA AAA CAT ATG ATT TTC TCC CTT CTC ATT CAC	Amplification of P_{ypqE}
**s9718**	AAA AAA CAT ATG CTG AAA AAA TTA TTC GG	Amplification of *ypqE*
**s9824**	P-GGG TGT TAG TAC GCC GTG	Amplification - The promoter of *ptsHI* from pKAM175
**s9825**	GGG TTA TCT AGG CTT GCG TCC C	Amplification - The *ptsG′* (EIICB) from pKAM175
**s9826**	GGG TTA TGT TTC TTC CGC AGC ATC	Amplification - The *ptsG′* (EIIC) from pKAM175
**s9827**	P-GGG AAA GGA GGT CAA TTC TTA TGG	Amplification - The *ptsG′* (EIIC) from pKAM175
**s9828**	CAT AAG AAT TGA CCT CCT TTA TGC TGC TGT TTC TTC CGC	Seperation of $EIIC^{Glc}$ from $EIIBA^{Glc}$
**s9829**	TAA AGG AGG TCA ATT CTT ATG CCT GGG AAA ACA GGT GAA	Seperation of $EIIC^{Glc}$ from $EIIBA^{Glc}$
**s9830**	AAG GCA GCA GCA GGG ATT	Fusion of P_{ptsG}-′*ptsG* (EIIA)
**s9831**	TCC CTG AGA AGA CTT GGT CCA TAA GAA TTG ACC TCC TCT TTT T	Fusion of P_{ptsG}-′*ptsG* (EIIA)
**s9832**	ATG GAC CAA GTC TTC TCA GGG A	Fusion of P_{ptsG}-′*ptsG* (EIIA)
**s9888**	GTA AGG GTT TTT GAC TCC GC	Complementation of *bglP*
**s9923**	ATT TCT GCA CTG ATT CTG G	Amplification of *ptsG*
**s9935**	GAC TTT CTT CAG GTT GGA G	Complementation of *licA*

**s9936**	GGA ATG CCG ATC GTC AT	Complementation of *licA*
**s9990**	AAA AAA GCT AGC ACCGGT CAC CCC TGC TCC TCC CGT	Amplification of P_{glcR}
**s9991**	AAA AAA CAT ATG CTC ATT CCT TTT CTC AGC A	Amplification of P_{glcR}
**s9994**	CGT GCA GTT CAG GAA TGT	Complementation of *gmuA*
**s9995**	CGG CAA ATT CGT TAA CAC	Complementation of *gmuA*
**s10595**	TTC ACC TGT TTT CCC CAT AAG AAT TGA CCT CCT CTT T	Mutation of *ptsG*
**s10596**	AGG AGG TCA ATT CTT ATG GGG AAA ACA GGT GAA GCA	Mutation of *ptsG*
**s10727**	TGG ATT ACC GTC TCA TCG	Complementation of *ptsG*
**s10728**	GTC GCA ATC ATA GGG AAC AT	Complementation of *ptsG*
**s10860**	CCC ATT CTG TAT GCA ATC C	Complementation of *gamP*
**s11135**	AAA AAA GCT AGC TGC CTC CGT TAT GAT TTC TT	Amplification of P_{ypqE}-*ypqE*
**s11136**	AAA AAA CCC GGG GTT GAA TAA AAA AAG CAA GG	Amplification of P_{ypqE}-*ypqE*
**s11137**	AAA AAA CCC GGG CCC ATG TTT AAT TCC TCC TC	Amplification of P_{yyzE}-*yyzE*
**s11138**	AAA AAA GCT AGC GAA TTG ATA AAT TAT ACC GTT ACA	Amplification of P_{yyzE}-*yyzE*
**s11244**	AAA AAA CCC GGG TGA CAA GCA CGG CGT ACT AAC	Amplification of *ptsG*
**s11245**	AAA AAA GCT AGC GTC ATT ATG CAC ACG GCT T	Amplification of P_{lev}-*levD*
**s11246**	AAA AAA GGA TCC ATA AAG CGG TCA TCA ATT CT	Amplification of P_{lev}-*levD*
**s11429**	CAG GCT CAC CAG CAA ATG AA	Complementation of *bglP*
**s11430**	GAG GGA ACG ACA AAT GAT AC	Complementation of *gamP*
**s11433**	GAC ATT GAG CGC TTT TAA G	Deletion of *licC*
**s11434**	GCG GCT CGA TAT TCT TGG A	Deletion of *licC*
**s11465**	AAA AAA AGA TCT GAG ATA AGG AGG TAT CAT GGG AGA ATC TAC	Amplification of *tkmA'*-his_6
**s11466**	AAA AAA CCC GGG AAG CTT TGT ACA ATG ATG ATG ATG ATG ATG ATT CGC AAT GGT GGA AAC TG	Amplification of *tkmA'*-his_6
**s11467**	AAA AAA TGT ACA GGG AAA ACA GGT GAA GCA GGA	Amplification of '*ptsG* (EIIBA)
**s11468**	TTT AAA CCC GGG TTA TTT TTC AAT CTT CAC AAT ATC T	Amplification of '*ptsG* (EIIBA)

**s11533**	AAA AAA GCT AGC ACC GGT ATA CGC GCA AAA CAC CTT TA	Amplification of P_{murQ}
**s11534**	CCC CCC CAT ATG GAT GCC CCC TGT TTT AAA A	Amplification of P_{murQ}
**s11543**	CCC CCC GGA TCC TTT CGC TTT TAT TGT AAA GAG	Amplification of *ypqE*
**s11569**	AAA AAA TGT ACA AAA GCT CCA GTT GCA AAA G	Amplification of *'gamP* (EIIBA)
**s11570**	AAA AAA CCC GGG CTT CAT TTG AAT GTG CTT TAG AT	Amplification of *'gamP* (EIIBA)
**s11571**	AAA AAA TGT ACA AAA GCC TTA AGT GAA GTC AAA	Amplification of *'bglP* (EIIA)
**s11572**	AAA AAA CCC GGG AGA TAA AGC AAG CAG CGC T	Amplification of *'bglP* (EIIA)
**s11573**	AAA AAA TGT ACA GAA TTT AAA AAA CTG ACA GAC ATT	Amplification of *'manP* (EIIA)
**s11574**	AAA AAA CCC GGG TTC GAT TTC TTC TAG CAG CT	Amplification of *'manP* (EIIA)
**s11616**	TAC GAT TGC TGC CGG AGC TCT TTT	The *sacX* seed sequence
**s11617**	AAA CAA AAG AGC TCC GGC AGC AAT	The *sacX* seed sequence
**s11618**	GGC CAA CGA GGC CGA TAT CAA CAA AGC GCG AGA	Deletion of *sacX*
**s11619**	TGA AAT CGA ATA AAT CCA GTT ACA AGC AAT TAA AAC CTC CTT	Deletion of *sacX*
**s11620**	AAG GAG GTT TTA ATT GCT TGT AAC TGG ATT TAT TCG ATT TCA	Deletion of *sacX*
**s11621**	GGC CTT ATT GGC CGA CTG ACC GTT GGT AGA ACC	Deletion of *sacX*
**s11697**	AAA AAA TGT ACA TTG CTG AAA AAA TTA TTC GGA	Amplification of *ypqE*
**s11698**	AAA AAA CCC GGG TTA TTT CGC TTT TAT TGT AAA	Amplification of *ypqE*
**s11808**	AAA AAA GGA TCC GAG ATA AGG AGG TAT CAT GCT GCA AAA TGA AAT CGG C	Amplification of *ptsG*
**s11809**	AAA AAA GGA TCC GAG ATA AGG AGG TAT CAT GAC AGC AGC ACC TGG GAA AAC A	Amplification of *ptsG*
**s11810**	AAA AAA TGT ACA CTG CAA AAT GAA ATC GGC	Amplification of *ptsG*
**s11856**	AAA AAA ACC GGT AAC CTT TAA GAT TTG CAT TC	Amplification of P_{sacX}
**s11811**	AAA AAA TGT ACA GAG ACC TTT ATT TAT CCG ATT	Amplification of *'gamP* (EIIA)
**s11857**	AAA AAA CTA GAA TTA AAA CCT CCT TTG CTG A	Amplification of P_{sacX}

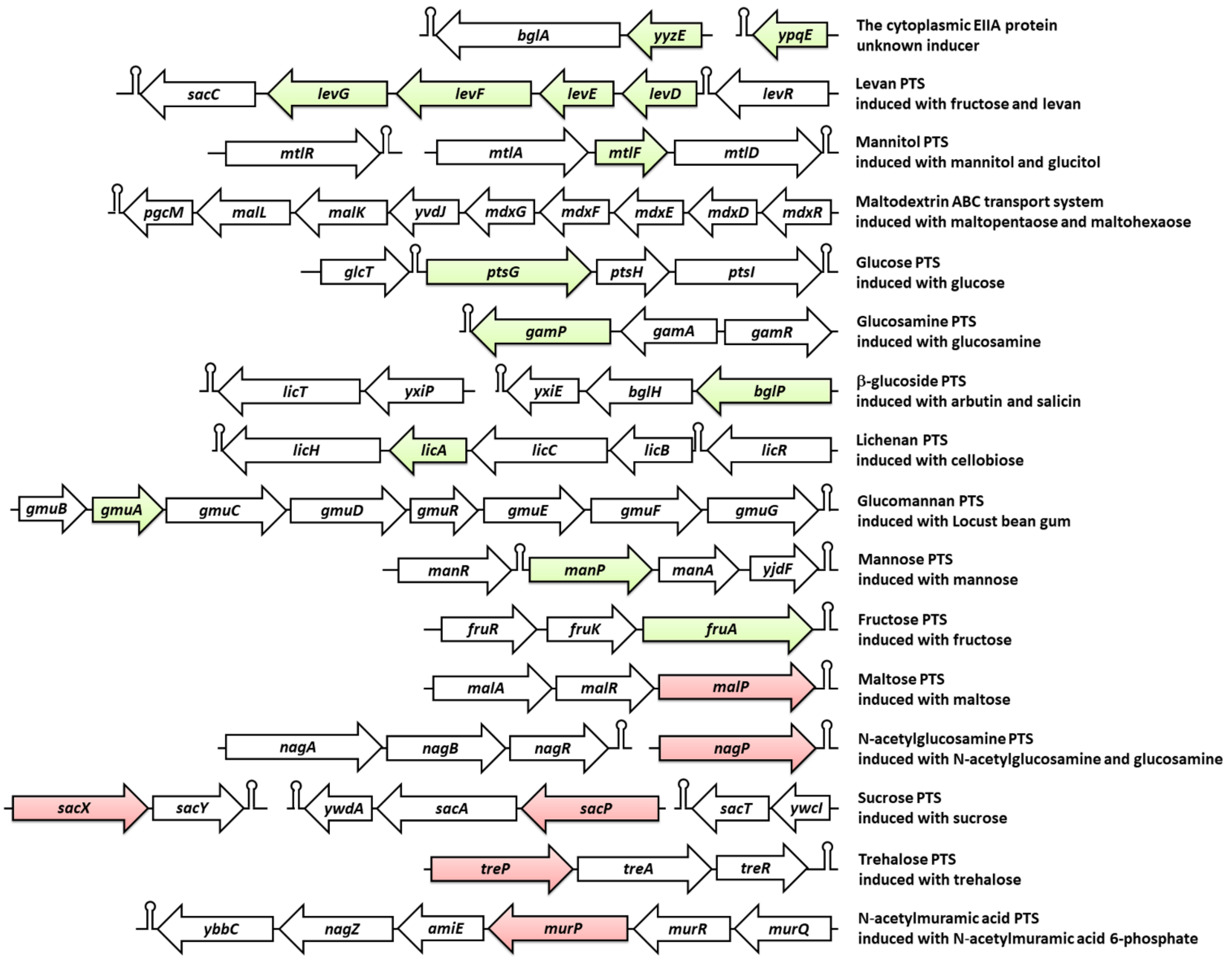

FIG S1 Genetic map of the PTS operons. The operons encoding the PTS transporters with (green) or without (red) EIIA domain are depicted. The maltodextrin operon is also shown.

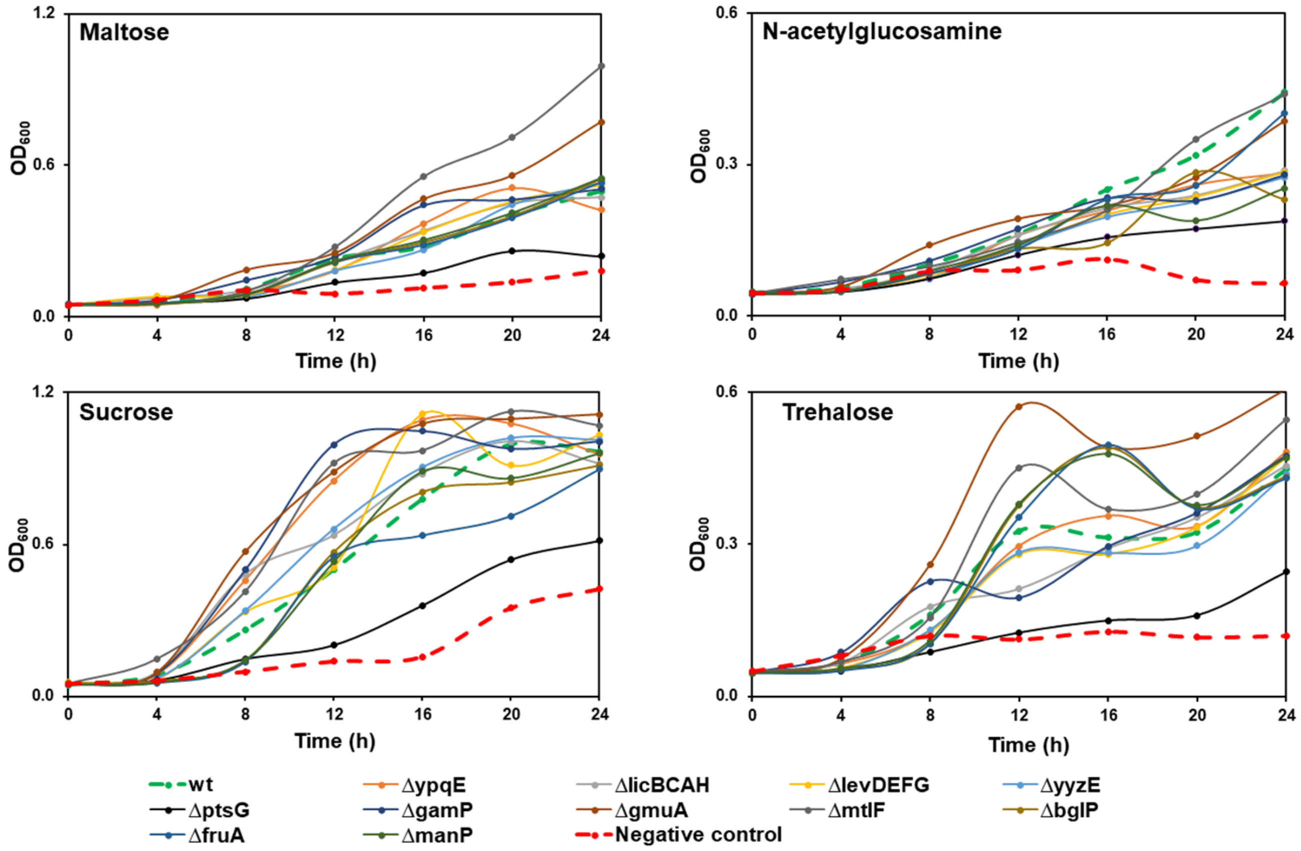

FIG S2 Growth of the EIIA single-deletion mutants in the presence of maltose, N-acetylglucosamine, sucrose and trehalose. Strains KM272 (Δ*ypqE*), KM285 (Δ*licBCAH*), KM287 (Δ*levDEFG*), KM288 (Δ*yyzE*), KM364 (Δ*ptsG*), KM366 (Δ*gamP*), KM373 (Δ*gmuA*), KM358 (Δ*mtlF*), KM423 (Δ*bglP*), KM435 (Δ*fruA*), KM646 (Δ*manP*) were cultured in Spizizen's minimal medium without citrate containing 0.5% (w/v) of maltose, GlcNAc, sucrose or trehalose as the sole carbon source. Strain KM0 (wt) was used as the positive control, while strains KM402 (Δ*manPA* Δ*mdxRDEFGyvdJmalKLpgcM* Δ*malP*), KM418 (Δ*nagP*), KM422 (Δ*sacP*) and KM338 (Δ*manPA* Δ*treP*) were used as negative control strains for maltose, GlcNAc, sucrose and trehalose, respectively.

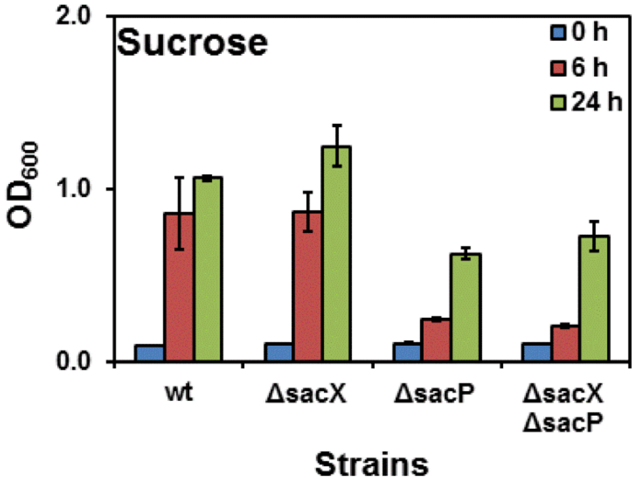

FIG S3 Deletion of the sucrose PTS transporters. The genes encoding the sucrose PTS transporters, namely *sacP* and *sacX*, were deleted and the growth of the mutants was investigated in the minimal medium containing sucrose as the sole carbon source in shaking flasks. Strains KM0 (wt), KM896 (Δ*sacX*), KM422 (Δ*sacP*) and KM908 (Δ*sacX* Δ*sacP*) were used for this study.

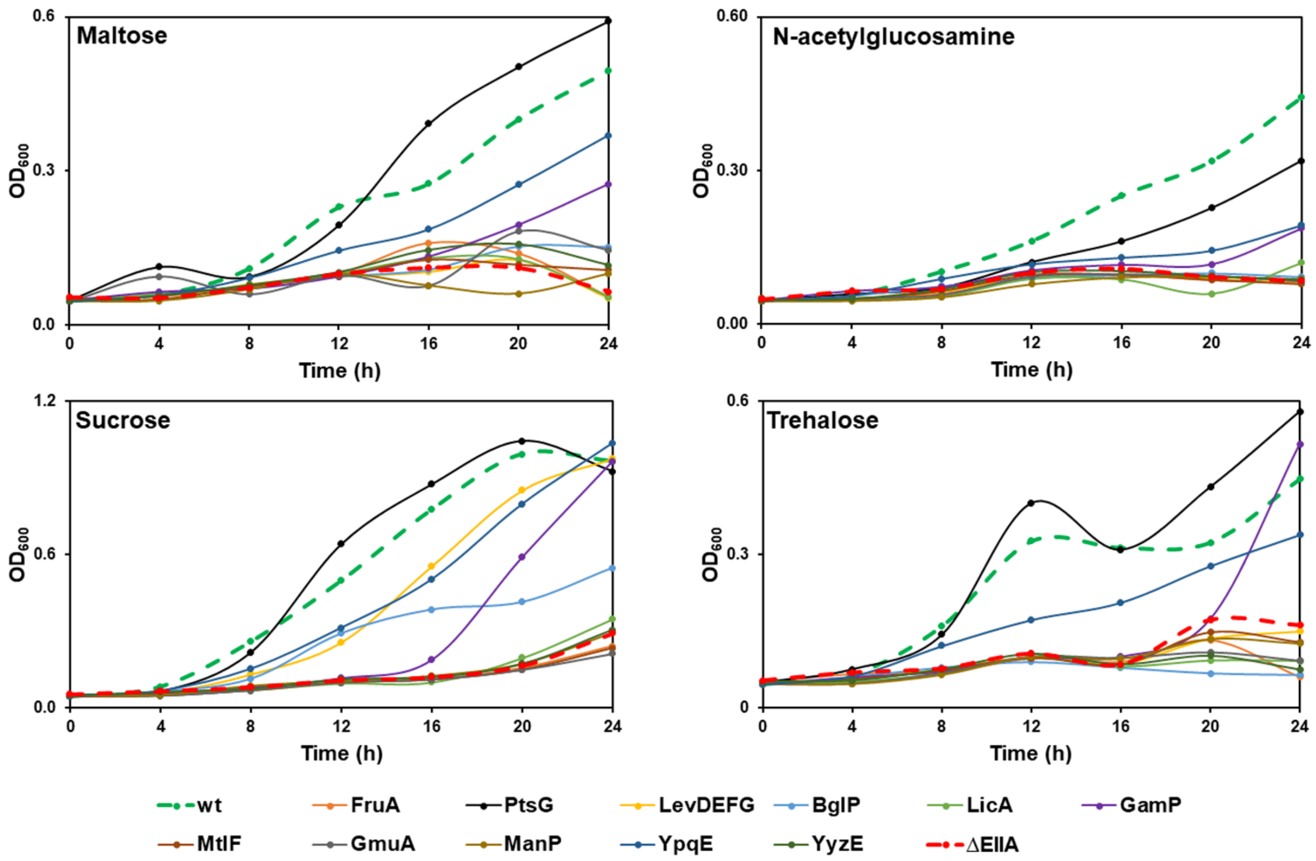

FIG S4 Growth of the strains having a single EIIA domain (or protein) in the ΔEIIA strain. Strains KM453 (KM455 *fruA*$^{+}$), KM790 (KM455 *ptsG*$^{+}$), KM791 (KM455 *levDEFG*$^{+}$), KM792 (KM455 *bglP*$^{+}$), KM793 (KM455 *licA*$^{+}$), KM794 (KM455 *gamP*$^{+}$), KM795 (KM455 *mtlF*$^{+}$), KM796 (KM455 *gmuA*$^{+}$), KM797 (KM455 *manP*$^{+}$), KM801 (KM455 *ypqE*$^{+}$), KM802 (KM455 *yyzE*$^{+}$) were cultivated in minimal media. Strain KM0 (wt) was used as the positive control, while strains KM455 (ΔEIIA) was used as negative control.

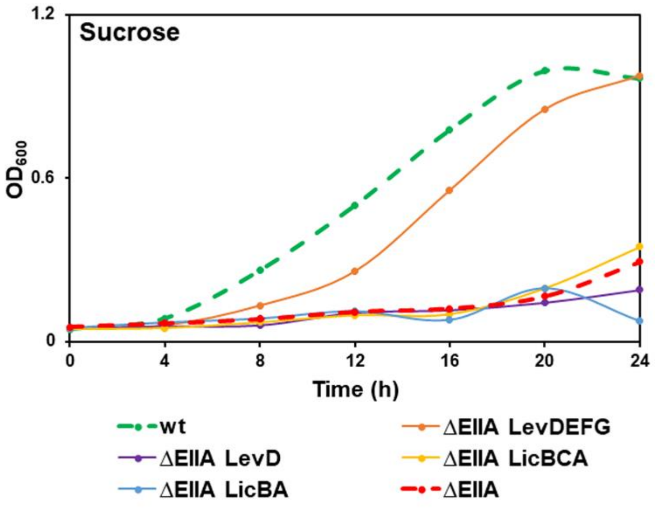

Fig. S5 Disabling the sucrose transport via LevDEFG and LicBCA complexes. Growth of strains KM791 (KM455 *levDEFG*$^{+}$), KM815 (KM455 *levD*$^{+}$), KM793 (KM455 *licBCA*$^{+}$) and KM820 (KM793 Δ*licC*) was investigated in minimal media. Strain KM0 (wt) and KM455 (ΔEIIA) were used as positive and negative controls, respectively.

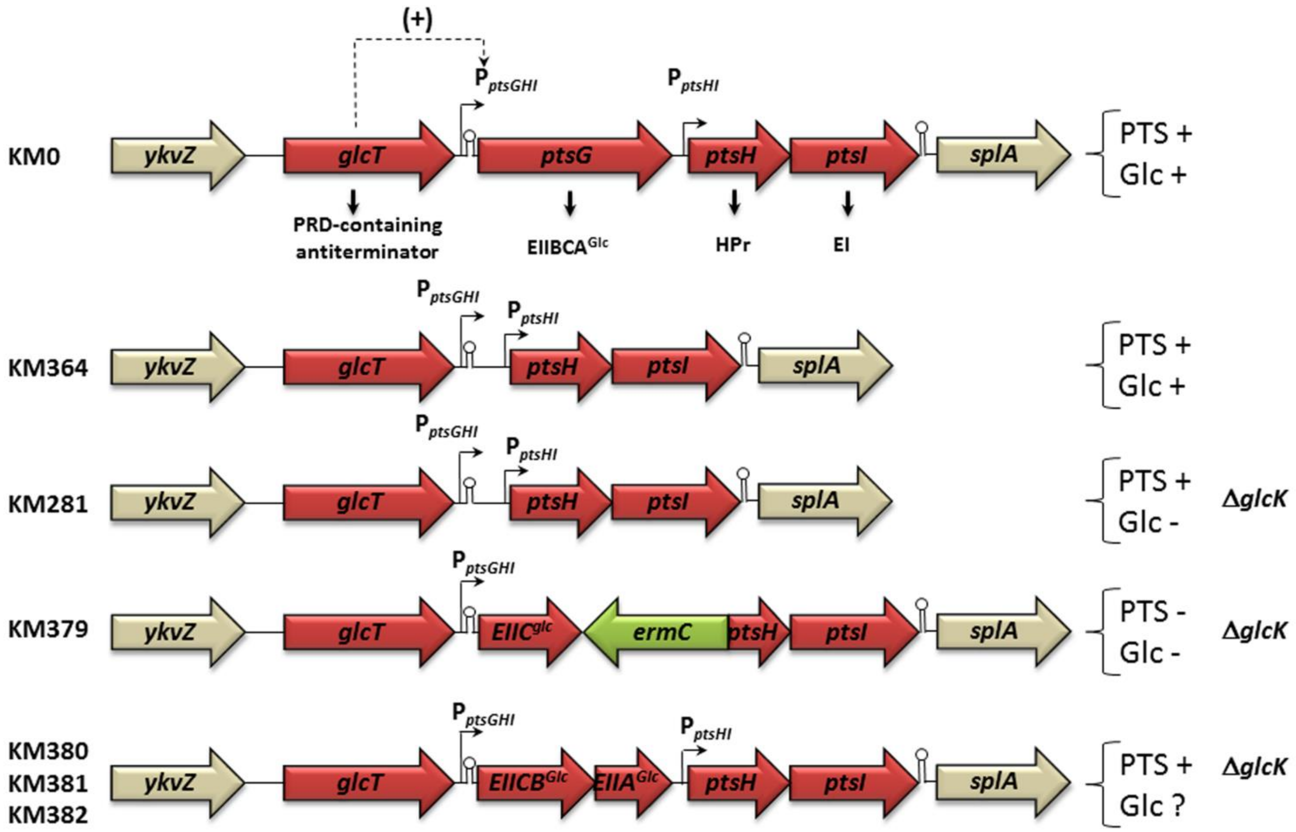

FIG S6 Genetic map of the strains containing the *ptsG* deletion or expressing truncated PtsG domains.

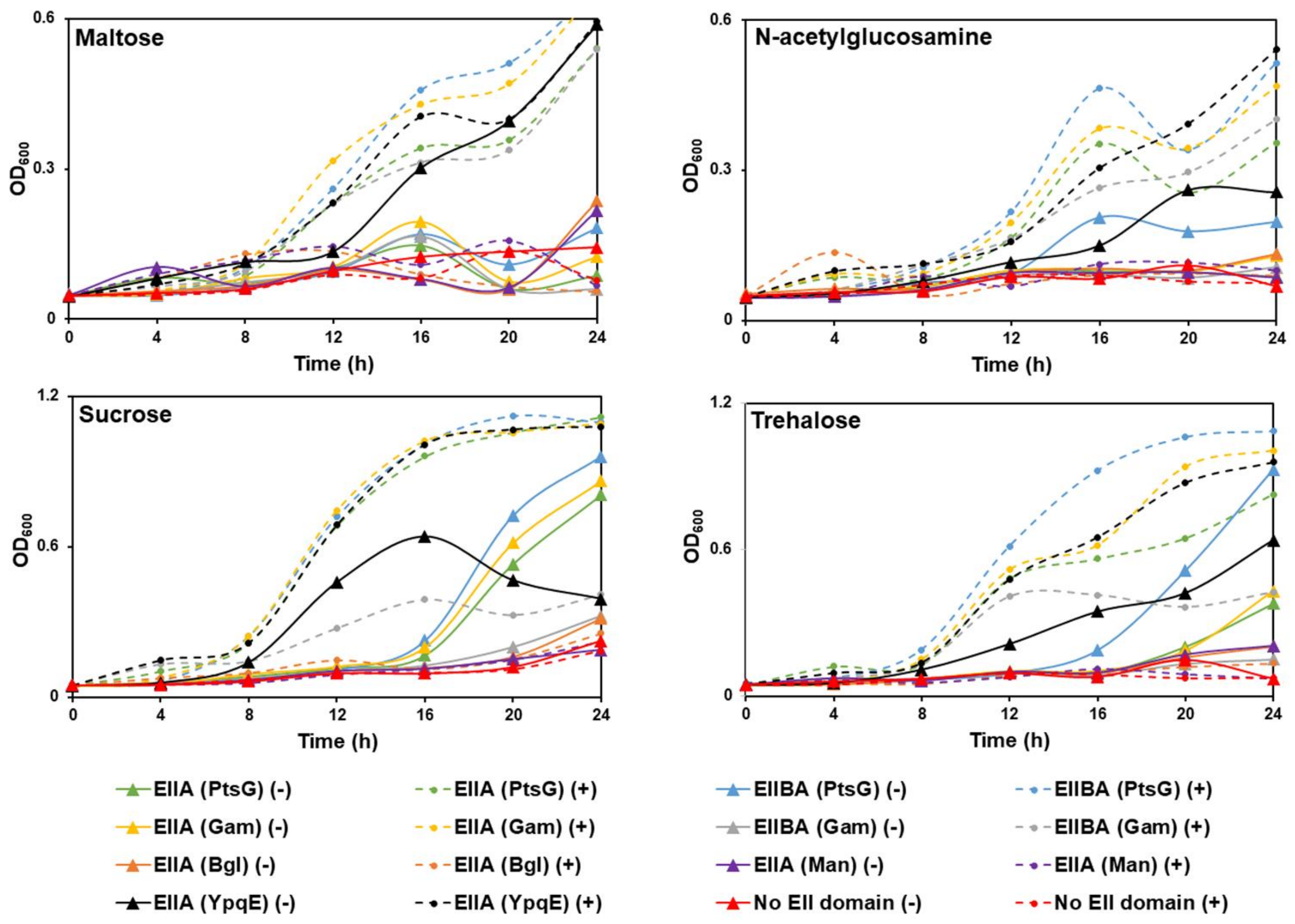

FIG S7 Interaction between the TkmA-fused proteins and the EIIA-deficient PTS transporters. Growth of the ΔEIIA strain containing TkmA-fused proteins in the presence of maltose, GlcNAc, sucrose and trehalose is shown. Strains KM870 (TkmA-His_6-$EIIBA^{Glc}$), KM884 (TkmA-His_6-$EIIBA^{Gam}$), KM885 (TkmA-His_6-$EIIA^{Bgl}$), KM886 (TkmA-His_6-$EIIA^{Man}$), KM887 (TkmA-His_6-$EIIA^{YpqE}$), KM873 (TkmA-His_6), KM916 (TkmA-His_6-$EIIA^{Glc}$) and KM917 (TkmA-His_6-$EIIA^{Gam}$) were cultured in Spizizen's minimal medium without citrate containing 0.5% (w/v) of maltose, GlcNAc, sucrose or trehalose as the sole carbon source. Experiment was carried out as explained in the legend of FIG 1 and IPTG was added upon inoculation to a final concentration of 1 mM.

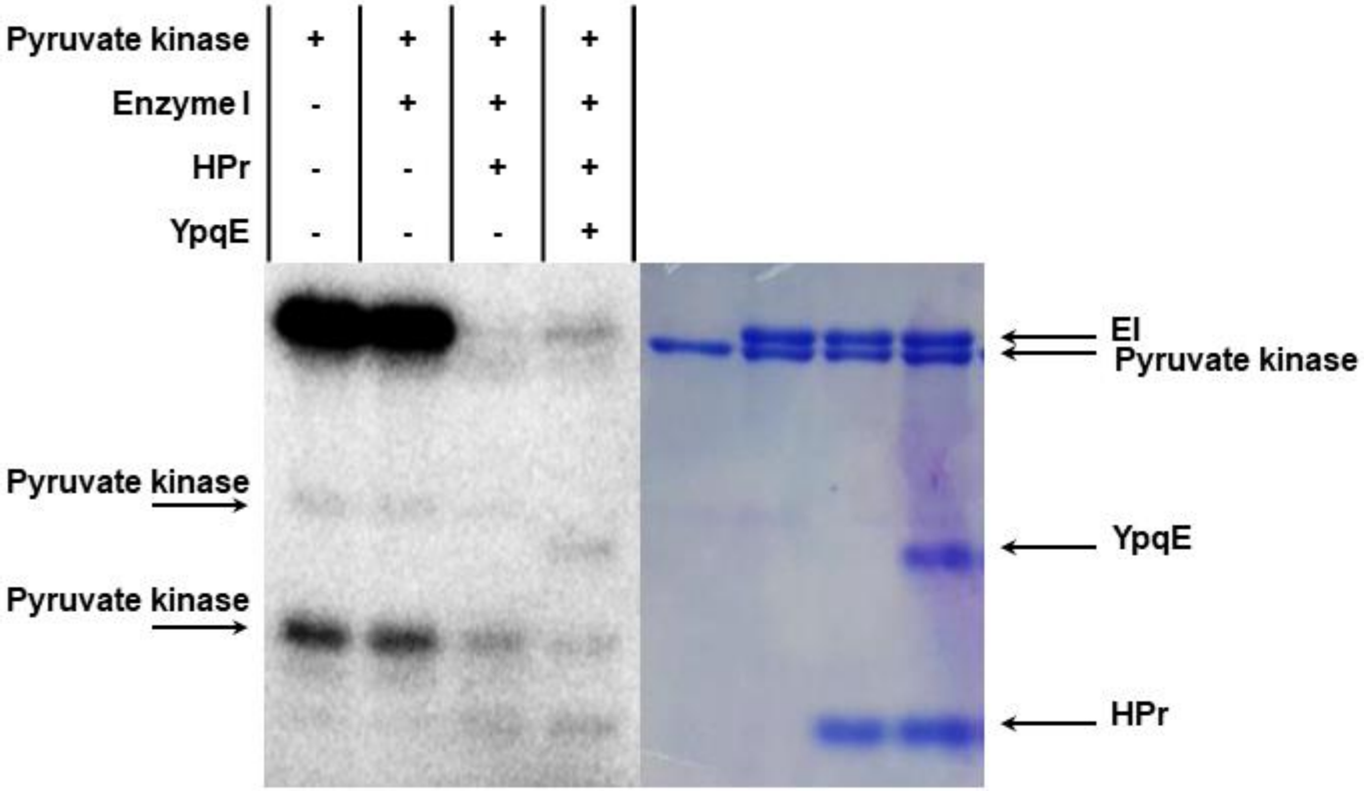

FIG S8 *In vitro* phosphorylation of YpqE by HPr(H15~P). Phosphorylation of YpqE using general proteins of PTS, enzyme I and HPr, and generated [^{32}P] PEP was carried out as described in materials and methods. [^{32}P] PEP was generated from [γ-^{32}P] ATP and pyruvate by pyruvate kinase. All reactions containing pyruvate kinase indicated unknown extra bands which was probably added by the producer for long-term storage of the enzyme.

MLKKLFGMGKIQEKVTEEVIYSPADGTVMDLSDVP-EIIA domain - PtsA (charge -2)

MGLFDKLKSLVSDDKKDTGTIEIIAPLSGEIVNIEDVP-EIIA domain - $EIIA^{Glc}$ (Crr) (charge -4)

EIIB domain- PEPKTSAQEEVGQQVEEVIAEPLQNEIGEEVFVSPITGEIHPITDVP-EIIA domain - PtsG (charge -9)

FIG S9 Secondary structure of the upstream region of the EIIA domains in PtsA and PtsG from *B. subtilis* and the $EIIA^{Glc}$ (Crr) from *E. coli*. The peptide properties including its charge (https://www.genscript.com/tools/peptide-property-calculator) as well as the peptide secondary structure (http://crdd.osdd.net/raghava//apssp/) were determined using the mentioned online softwares. The yellow highlight indicates the residues forming the α-helix at the N-terminus.

References

1. Yanisch-Perron C, Vieira J, Messing J. 1985. Improved M13 phage cloning vectors and host strains: nucleotide sequences of the M13mp18 and pUC19 vectors. Gene 33:103-19.
2. Baba T, Ara T, Hasegawa M, Takai Y, Okumura Y, Baba M, Datsenko KA, Tomita M, Wanner BL, Mori H. 2006. Construction of *Escherichia coli* K-12 in-frame, single-gene knockout mutants: the Keio collection. Molecular Systems Biology 2:1-11.
3. Koo BM, Kritikos G, Farelli JD, Todor H, Tong K, Kimsey H, Wapinski I, Galardini M, Cabal A, Peters JM, Hachmann AB, Rudner DZ, Allen KN, Typas A, Gross CA. 2017. Construction and Analysis of Two Genome-Scale Deletion Libraries for *Bacillus subtilis*. Cell Syst 4:291-305 e7.
4. Rahmer R, Morabbi Heravi K, Altenbuchner J. 2015. Construction of a super-competent *Bacillus subtilis* 168 using the P_{mtlA}-*comKS* inducible cassette. Frontiers in Microbiology 6.
5. Bolivar F, Rodriguez RL, Greene PJ, Betlach MC, Heyneker HL, Boyer HW, Crosa JH, Falkow S. 1977. Construction and characterization of new cloning vehicles. II. A multipurpose cloning system. Gene 2:95-113.
6. Guérout-Fleury A-M, Frandsen N, Stragier P. 1996. Plasmids for ectopic integration in *Bacillus subtilis*. Gene 180:57-61.
7. Motejadded H, Altenbuchner J. 2007. Integration of a lipase gene into the *Bacillus subtilis* chromosome: Recombinant strains without antibiotic resistance marker. Iran J Biotechnol 5:105-109.
8. Altenbuchner J, Viell P, Pelletier I. 1992. Positive selection vectors based on palindromic DNA sequences. Methods Enzymol 216:457-66.
9. Hoffmann J, Bóna-Lovász J, Beuttler H, Altenbuchner J. 2012. *In vivo* and *in vitro* studies on the carotenoid cleavage oxygenases from *Sphingopyxis alaskensis* RB2256 and *Plesiocystis pacifica* SIR-1 revealed their substrate specificities and non-retinal-forming cleavage activities. Febs Journal 279:3911-3924.
10. Wenzel M, Altenbuchner J. 2015. Development of a markerless gene deletion system for *Bacillus subtilis* based on the mannose phosphoenolpyruvate-dependent phosphotransferase system. Microbiology 161:1942-9.
11. Altenbuchner J. 2016. Editing of the *Bacillus subtilis* genome by the CRISPR-Cas9 system. Appl Environ Microbiol 82:5421-7.
12. Heravi KM, Wenzel M, Altenbuchner J. 2011. Regulation of *mtl* operon promoter of *Bacillus subtilis*: requirements of its use in expression vectors. Microb Cell Fact 10:83.

13. Graf N, Wenzel M, Altenbuchner J. 2016. Identification and characterization of the vanillin dehydrogenase YfmT in *Bacillus subtilis* 3NA. Applied Microbiology and Biotechnology 100:3511-3521.

14. Wenzel M, Altenbuchner J. 2013. The *Bacillus subtilis* mannose regulator, ManR, a DNA-binding protein regulated by HPr and its cognate PTS transporter ManP. Mol Microbiol 88:562-76.

15. Norrander J, Kempe T, Messing J. 1983. Construction of improved M13 vectors using oligodeoxynucleotide-directed mutagenesis. Gene 26:101-6.

16. Bisicchia P, Botella E, Devine KM. 2010. Suite of novel vectors for ectopic insertion of GFP, CFP and IYFP transcriptional fusions in single copy at the *amyE* and *bglS* loci in *Bacillus subtilis*. Plasmid 64:143-149.

17. Heravi KM, Altenbuchner J. 2014. Regulation of the *Bacillus subtilis* mannitol utilization genes: promoter structure and transcriptional activation by the wild-type regulator (MtlR) and its mutants. Microbiology 160:91-101.
